# Boosting vegetation, biochemical constituents, grain yield and anti-cancer performance of cultivated oat (*Avena sativa* L) in calcareous soil using oat extracts coated inside nanocarriers

**DOI:** 10.1186/s12870-022-03926-w

**Published:** 2022-11-24

**Authors:** Noura E. Mahmoud, Asmaa A. Mahdi, Ashraf M. A. Barakat, Reda M. Abdelhameed

**Affiliations:** 1 Biochemistry Unit, Plant Genetic Resources Department, Desert Research Center, Cairo, Egypt; 2 Zoonotic Diseases Department, National Research Centre, 33 Bohouth Str. Dokki, Giza, Egypt; 3 Applied Organic Chemistry Department, Chemical Industries Research Institute, National Research Centre, Scopus Affiliation ID 60014618, 33 EL Buhouth St., Dokki, Giza, 12622 Egypt

**Keywords:** Cu-BTC MOF, Separation, Salicylic acid, anthraquinone, and triacylglycerol, Oat plant

## Abstract

Calcareous soil contains many problems such as the lack of sources of major and minor elements that are useful for plant growth and development. Plant extracts and nanoparticles are very popular as biostimulants in plant production. Here, the effect of aqueous, non-aqueous and alcoholic oat extracts on the growth, biochemical response of oats leaves and grains grown in experimental fields under new reclamation lands were studied. Moreover, different oat extracts were a pathway through the copper-dependent metal–organic framework (MOFs) to separate bioactive molecules from extracts such as salicylic acid, anthraquinone, and triacylglycerol. Additionally, the separated molecules incorporated in Cu-BTC MOFs and oats extracts missed active molecules were spray applied on oat plants. The results showed that the treated plants showed stimulatory responses in growth and physiology. The treatments improved plant growth and biomass, enhanced total protein, water-soluble carbohydrates, free phenolic compounds content in oat leaves, photosynthesis, and chlorophyll contents. The treatments also improved the level of vitamins E and K, phenolic compounds, and avenanthramides C in the oat grains. Moreover, the treatments showed an improvement in the yield of oats (grain and straw) using water and alcoholic oat extracts in which the active molecules were missed. Our findings demonstrate that Cu-BTC and oats extracts can act as a biostimulant to enhance the biological and chemical properties of oats and increase the yield in calcareous soils. The cytotoxicity study of oats (produced from AE, c@Cu-BTC, and AE-c treatments) was conducted using Vero Cell lines. The anticancer activities of different oat grains were carried out using MCF 7cell lines. The results show that the grains produced from the AE, c@Cu-BTC, and AE-c treatments possessed 94.3, 72.3, and 100% activity towards the cancer cell line. Removal of growth inhibitors from spray solutions increases grain yield and anticancer activity.

**Supplementary Information:**

The online version contains supplementary material available at 10.1186/s12870-022-03926-w.

## Introduction

Egyptian calcareous soils constitute about 25–30% of the total area, according to the estimates of the Ministry of Agriculture [1]. The basic composition of calcareous soils is calcium carbonate (CaCO_3_), which is very complex for plant growth because it is impermeable to water and plant roots. When phosphorous fertilizers are added to calcareous soils, a series of stabilization reactions occur that gradually reduce its solubility and ultimately its availability to plants. The northwestern coast of Egypt is considered one of the important areas for its capabilities and its wonderful location. Significant efforts have been made to assess the agricultural potential to meet the nutritional needs of the growing population of Egypt. In calcareous soils where the pH is high and mostly calcium carbonate; plants have too little phosphorous and potassium which causes more serious problems than a deficiency. Increasing the availability of these nutrients is one of the important goals in plant nutrition [2]. Calcareous soils contain high levels of calcium carbonate (CaCO_3_) that influence soil properties related to plant growth, such as soil–water relationships and the availability of plant nutrients [3]. Therefore, we select these specific areas to investigate our topic towards the cultivation of oats crops.

Oats (*Avena sativa* L.) is one of the most important cereal crops in the world due to their use as a major source of vital nutrients for both humans and animals. It is cultivated on an area of approximately 10 million hectares in cold regions around the world. It has been used as a medicinal and food plant to treat humans and animals [4, 5]. The authors describe the essential medical role of *Avena sativa* in diseases as diverse as inflammatory diseases and cardiovascular disease [6]. *Avena sativa* has an advantage because it is contained proteins, avenanthramides, lipids, beta-glucan, alkaloids, flavonoids, triterpenoid saponins, and sterols. Oat extract can be applied in pharmacological including anti-inflammatory, antioxidant, immunomodulatory, antidiabetic, gastrointestinal, hypolipidemic, neuroprotective, cardiovascular, and it also has application in other biological activities [7]. In addition, oats were also rich in body building nutrients including silicon, manganese, zinc, calcium, phosphorous, vitamins A, B1, B2, and E. Thus, oat meal, which contains 15% gluten, is suitable for celiac patients [8]. Oats have antioxidants such as vitamin E, flavonoids, and phenolic acid compounds such as avenanthramides. Being antioxidants, they prevent free radical damage to DNA, RNA, proteins, and cellular organelles by enhancing SOD activity, DPPH radical scavenging activity, and reducing the level of MDA [9–11].

Avenanthramides, a group of polyphenol alkaloids, is also unique to *Avena sativa* L. Most of the anticancer properties of the avenanthramides group come from blocking reactive species. Cell proliferation is blocked and inhibition of epithelial and mesenchymal transformation and metastasis. Avenanthramide is promising as chemopreventive and anticancer phytochemicals. Oats β-glucan as water-soluble fiber caused a significant reduction in total cholesterol, decreased lipoprotein levels, calories, body weight, blood pressure, and blood glucose control, thus having a hypoglycemic response, anti-obesity, and cardioprotective effect [12]. Regarding plant extracts, ethanolic oat extract is considered to have antimicrobial effects as positive and negative antibacterial, and antifungal activity [6]. In this direction, oat extracts contain many chemical compounds that differ in their properties from each other. Some of them have an inhibitory effect and others have a stimulating effect on plant growth and improving its chemical properties, and they are called allochemical compounds.

Allelochemicals can be used as growth regulators, herbicide, insecticide, and antimicrobial crop protection products [13]. Several of these phenolic compounds have been identified as allelochemicals in other grain species [14, 15]. Phenolic compounds are one of the main groups of substances involved in the allelopathy of wild oat root [16]. Phenolic acids like coumaric acid, vanillic acid, and ferulic acid can be found in Wild oats, as well as other phenolic compounds [17]. However, the isolation and identification of chemicals from plants with biological activity is not indicative that these compounds interfere in nature through allelopathy [18]. Enhancing the activity of allelochemicals was studied for the first time in this study by loading them into novel nanocomposites such as MOFs.

MOFs are a type of material with adjustable pore sizes formed through the self-assembly of organic bonds coordinated into metal ions or groups. MOFs have excellent properties, which include excellent adsorption capacity, customized shape, and size, hierarchical structure, many surface active sites, high specific surface areas, high chemical stability, easy modification, and operation [19]. MOFs have shown great potential for environmental remediation adsorption and fuel purification [20]. MOFs are efficient for the removal of harmful gases, through specific interactions between harmful adsorbents and the host. Moreover, MOFs contain multiple functional groups, pair bonds, and metal ions that can help in understanding the interactions between MOFs and contaminants and thus increase the efficiency of adsorption. MOF has been used in many fields such as application as fertilizer [21–23]. In addition, ethylene diaminetraacetic-MOFs (Fe-MOF-EDTA) were used as iron sources in *Phaseolus vulgaris,* compared to other Fe sources, Fe-MOF-EDTA caused a 9.6% increase in plant weight and improved chlorophyll, protein, and enzyme activities [24]. MOFs contain the elements that crops need as nutrients, such as nitrogen and phosphorus, and possibly essential mineral micronutrients, such as iron, zinc, etc. [25, 26]. During the wheat growth period, MOFs degraded by 50.9%, and the rate of decomposition was closely related to soil temperature. It was also found that the rate of decomposition increases with increasing soil temperature. Moreover, the nutrient concentration in the soil indicates that MOF has stable nutrient release efficiencies and can provide a continuous supply of nutrients throughout the growth period of wheat, which showed a great role of MOF as a fertilizer that is beneficial in agricultural production and environmental protection [27]. The aim of this study is to evaluate the different effects of oat extracts loaded with nano-components of MOFs on the biochemical content of oats grown under North Coast conditions and their effect on the properties of oats.

## Material and methods

### Chemicals and materials

Copper (II) nitrate trihydrate (> 99%) and N, dimethylformamide (DMF, 99.9%) was obtained from Aldrich, USA, Benzene-1,3,5-tricarboxylic acid was purchased from Merck, Darmstadt-Germany. Solvents, chloroform, toluene, ethyl acetate, and hexane were used as received without any purification. The chemicals used for the sample preparations were of analytical reagent grade. Standards for α-tocopherol (vitamin E) and phylloquinone (vitamin K) were purchased from Sigma (St Louis, MO, USA). Methanol, ethanol, acetonitrile, diethyl ether, and petroleum benzene were obtained from Fisher (Waltham, MA, USA). Oat seeds used in this study were provided from the Institute of Field Crops, Agricultural Research Center, Giza, Egypt. The chemical analysis of soil and water is presented in Table (S1). The meteorological data for the Maryout location are presented in Table (S2).

### Preparation of Cu-BTC

The Cu–BTC MOF was made as follows 2.077 g of copper (II) nitrate trihydrate and 1.0 g of benzene-1,3,5-tricarboxylic acid were dissolved in 50 mL of DMF. Then, the solution mixture was heated at 160 °C in the open air until the complete evaporation of the DMF. The reaction vessel was cooled to room temperature and the solid Cu-BTC was isolated by centrifugation and washed with ethanol five times to remove unreacted materials. The Cu-BTC product was then dried in an oven at 105 °C overnight, and then stored in a vacuum until used [28].

### Preparation of oats (*Avena sativa* L.) grain extracts

#### Hexane and methanol extraction

Oat grains (150 g) are gently crushed, loaded into a porous cellulose cap, which is placed inside the Soxhlet extractor. Then, the solvent (500 mL n-hexane or methanol) was added to a round bottom flask connected to a Soxhlet extractor and thickener. The solvent is heated using a water bath heater and its vapors pass through the apparatus to the condenser. The condensed liquid is then dripped into the tank containing the cellulose cap, and after the tank is filled, the solvent returns to the flask. The process was run for 3 days. Once the process was over, hexane or ethanol was evaporated using a rotary evaporator, leaving a small crop of the extracted plant matter (about 10 to 15 mL) in a glass jar. The components in the hexane extract were determined using GC/MS mean while methanol extract was determined using HPLC [29, 30].

#### Aqueous extraction

Oat grains were extracted with aqueous extraction according to previous method [31] as follows: the oat grains (150 g) were milled into flour samples. The aqueous extraction process was carried out at temperatures of 85 °C for three hours, and pH values of 7.1. The extract was evaporated to dryness and the residues were kept in deep freezer until uses. The constituents in the aqueous extract were identified using HPLC.

### MOFs separation studies

The three extract sources (hexane, methanol and water) were added to Cu-BTC MOF (40 mg), separately. The samples were shaken at 30 °C using a temperature-controlled shaker for 24 h. The liquid solution was separated from the adsorbent particles by centrifugation at 5000 rpm for 10 min and analyzed using GC/MS and HPLC.

### Sample characterization

#### X-ray analysis

Powder X-ray diffraction (XRD) patterns were examined for Cu-BTC, a@Cu-BTC, b@Cu-BTC and c@Cu-BTC on a copper-connected X'Pert MPD Philips diffractometer. K_α_ monochromatic. A new composite materials scanning electron microscope was measured on a Hitachi SU-70-JP microscope. GC–MS analysis was used to determine the component of the oat extract. Run with GC/MS Finnigan Mat SSQ 7000, EI 70 eV. operating conditions; DB-5 capillary column, 30 m × 0.25 mm ID [(5%—vinyl) methyl polysiloxane]. Analysis was performed at programmed temperature: initial temperature of 50 °C for 0 min, then increased at a rate of 5 °C/min, until it reached 300 °C (keep for 5 min). The injector temperature was set at 250 °C and the detector temperature at 280 °C. Helium was used as carrier gas at 1 mL/min, injection volume was 2 μL, and injection mode was split-free. The compounds were identified by matching their MS with those recorded in the MS Library (Wiley) and comparing them with those of the reference compounds [32].

#### High performance liquid chromatography (HPLC)

HPLC analysis was performed using an Agilent HPLC system (1100 infinity series) coupled with a diode array detector (DAD) and a fluorescence detector. Compounds were separated using a 5 µM Agilent Zorbax Eclipse XDB 250 × 4.6 mm, 5 µM particle size C18 column fitted with a C18 protection cartridge that was maintained from 22 to 40 °C. The mobile phase programs were firstly, 0.05 M phosphate buffer (A) at pH 2.4 and methanol (B) with the following gradient: 5–60% B in 10 min; 60–90% B in 6 min. The flow was set at 0.6 mL/min, 35 °C column temperature and quantitated at the wavelength of 350 nm to separate avenanthramide-C as described by [33]. Secondly, the solvent system for free phenolic acids separation was a gradient of water acetic acid (A, 0.1%) and methanol (B). The gradient used starts with 95% A, 95 to 50% for 10 min, 50 to 30% for 5 min, then 30 to 10% for 5 min at a flow rate of 1.0 mL/min, 40 °C column temperature. The detector at the wavelength of 280 nm. as previous work [34]. Third, the mobile phase consists of 85% methanol, 2-propanol 9%, 5% acetonitrile, and 1% methanol solution. The flow rate of the mobile phase was 0.8 ml/min, the column was operated at 22 °C with fluorescence detection was carried out at an excitation wavelength of 246 nm and an emission wavelength of 430 nm for the separation of vitamins K [35]. Finally, mobile phase was methanol, ethanol and petroleum benzene (isocratic elution) and the flow rate was 1.0 mL/min, and column was cartridge maintained at 35 °C with quantification at wavelength 292 nm for vitamin E [36]. The injection volume was 20 μL for each analysis. The results were calculated with the help of peak areas for internal standard solutions. Laboratory solution software provided by Shimadzu was used to evaluate the chromatogram.

### Field experiment

Field experiments were conducted during the 2019/2020 and 2020/2021 seasons at the Desert Research Center (DRC) Agricultural Experimental Station in Maryout Station, Egypt. The experiments were designed in Randomized Complete Block Design with three replicates. All treatments including the collection of plant material were performed in accordance with the Desert Research Center (DRC, Egypt) guidelines and regulations. Experiments were conducted to evaluate the different effects of oat extracts loaded within MOFs on the biochemical content of oats grown under Maryout conditions. The area of the experimental unit was 4 m^2^ (2 m × 2 m) and the recommended fertilization for this soil type was applied according to the Desert Research Center with supplemental irrigation (drip-irrigated at a rate of 4 L in an hour for a half-hour per week). The experiment included eleven treatments with concentration 20 mg L^−1^ (Table 1).

**Table 1:** The experimental design of foliar applications treatments codes

No	Abbreviations	Foliar applications treatments
1	Control	Control
2	Cu-BTC	Copper based MOFs
3	HE	Hexane extract
4	ME	Methanol extract
5	AE	Aqueous extract
6	a@Cu-BTC	Copper based MOF incorporated with separated molecule from hexane extract
7	b@Cu-BTC	Copper based MOF incorporated with separated molecule from methanol extract
8	c@Cu-BTC	Copper based MOF incorporated with separated molecule from aqueous extract
9	HE-a	Hexane extract after removed molecule captured with MOF
10	ME-b	Methanol extract after removed molecule captured with MOF
11	AE-c	Aqueous extract after removed molecule captured with MOF

Foliar applications were applied twice after 30 and 60 days from sowing. The spray volume was 1000 L ha^−1^ using Tween 20 as a wetting agent at 0.05%. Fresh leaves samples were taken randomly from each treatment at 75 days from sowing and tested for growth traits, samples are kept in a deep freezer at -20 °C until photosynthetic pigments determined. The dried leaves was ground and measured water-soluble carbohydrate, free phenolic compounds and total protein. Also, plants were harvested after 170 days from sowing to determine grains and straw yields (ton ha^−1^) as well as determination of the active substances in the grains as free and polyphenolic acids compounds and fat-soluble vitamins i.e. E and K.

### Anti-cancer activity

For anti-cancer activity, the MCF 7cell line was used. The cytotoxicity of grains was tested against vero cell line. At 37 °C in a 5% CO_2_ incubator, all cell line was cultured in a minimum essential medium (MEM, GIBCO) fortified with 2 mL-glutamine, 4.5 g/L glucose, and 5% foetal bovine serum FBS as growth media. Three different grain sources were used, including AE, c@Cu-BTC and AE-c in different concentrations, i.e., 12.5, 25, 50, 100 µg/mL.

### Statistical analysis

Data were subjected to one-way ANOVA and differences between means were determined at the 5% probability level using Duncan's new multiple range test. SPSS Soft, version 16 (SPSS, Richmond, USA) was used [37].

## Results

**Fig. 1:**
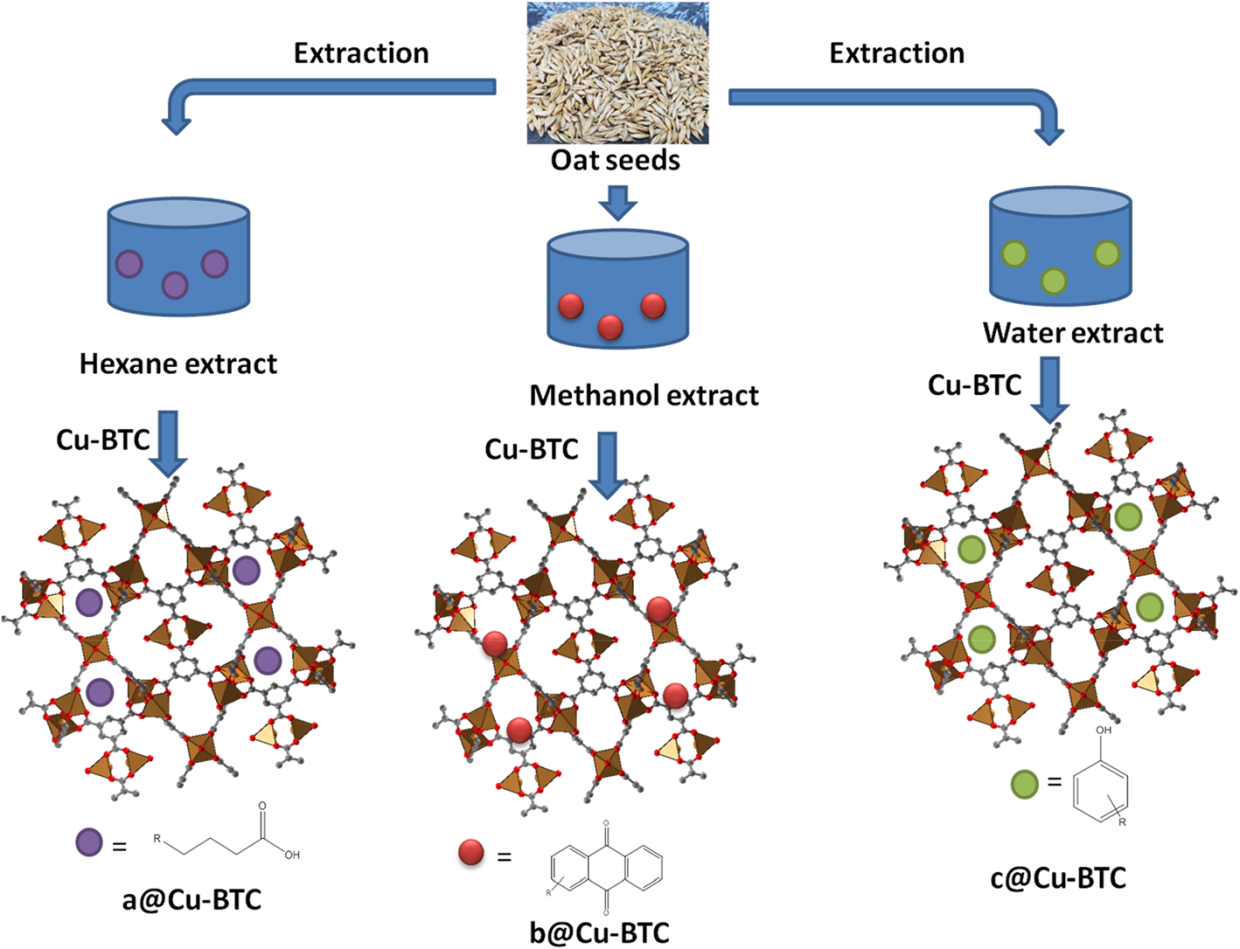
Schematic diagram for extraction and separation of bioactive compounds from oat extract, incorporation of extract with Cu-BTC

The present work focused on two novel approaches. The first approach deals with the separation of bioactive compounds from oats extract using Cu-BTC (Fig. 1). The second one is the application of crude extracts, incorporated bioactive molecule onto Cu-BTC and the oat extract after separation of bioactive molecules in evolution of the growth parameters and bioactive compounds ratio on oat plant.

### Separation and identification of compounds of different extracts

Figure S1 shows the GC–MS of oat hexane extracts. The chromatogram was checked with library and it produced that the extract has chemicals like phytosterol fatty acyl esters, triacylglycerol, free linoleic acid, γ-tocopherol, free phytosterols, and ferulate phytosterol esters. The important notification is disappearing of peak at RT = 16.43 min, when the residual hexane extract after path through Cu-BTC MOF was injected in the GC machine (Figure S2). This peak is related to triacylglycerol. Figures S3 and S4 show the HPLC chromatograms of methanol extract of oat grains and methanol extract of oat grains after path through Cu-BTC, respectively. According to references in HPLC injection, the chemicals may be found in the oat methanol extract were benzoquinone, anthraquinone and complex quinones; amino acids and peptides; alkaloids and cyanohydrins; sulfide and glucosinolates; and purines and nucleosides. The key notification is disappear of peak at RT 11.15 min. this peak is related to anthraquinone. The aqueous extract of oat grain was also path through Cu-BTC MOF and both extracts and residual extracts after path were injected in HPLC machine (Figure S5 and S6). The compounds found in the extract was water-soluble organic acids like straight chain alcohols, aliphatic aldehydes, and ketones; simple unsaturated lactones; polyacetylenes, benzoic acid and its derivatives; cinnamic acid and its derivatives; salicylic acid, gibberellic acid, coumarin; flavonoids, tannins, terpenoids and steroids. The nice finding is disappearing of peak at RT 3.31 min. when the water extract path through Cu-BTC MOF. This peak is related to salicylic acid.

### X-ray diffraction patterns and SEM analysis

**Fig. 2:**
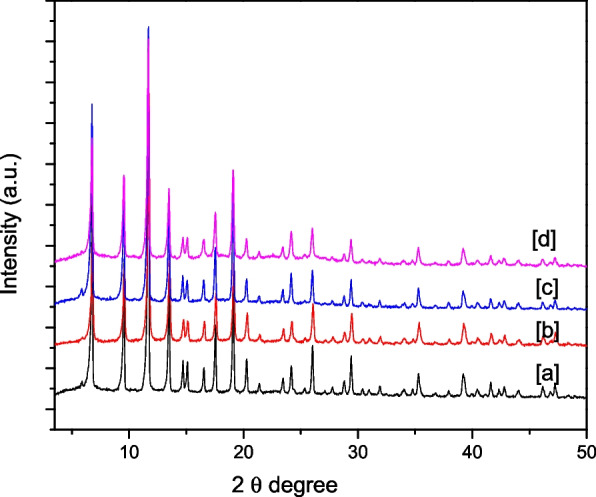
PXRD patterns for **a** Cu-BTC, **b** a@Cu-BTC, **c** b@Cu-BTC and **d** c@Cu-BTC

Figure 2 shows powder X-ray diffraction (PXRD) of Cu-BTC, and Cu-BTC after immobilization of bioactive compounds on its pores. The obtained data were matched with the powder X-ray diffraction of commercial Cu-BTC, and the results clearly showed that Cu-BTC MOF retained its crystal and possesses the structure of Cu-BTC.

**Fig. 3:**
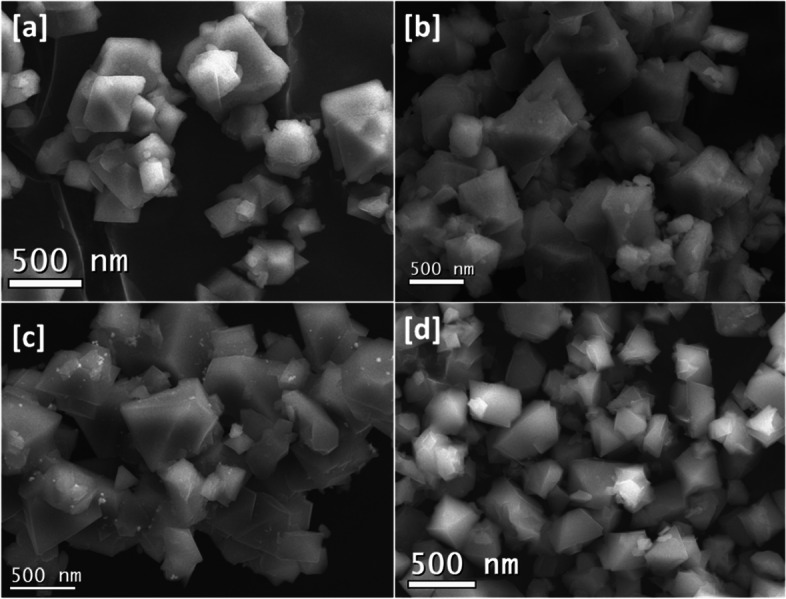
SEM image for **a** Cu-BTC, **b** a@Cu-BTC, **c** b@Cu-BTC and **d** c@Cu-BTC

The topographical features of the MOFs were characterized by the surface of the MOFs examined using electron microscopy. Electron micrographs presented in Fig. 3 illustrate Cu-BTC crystals of pyramidal shape and dimensions in the range of 300–500 nm.

### Effect of Cu-BTC, and oats extracts on *Avena sativa* L.

Foliar spraying of oats with oats extracts, Cu-BTC, and oats extracts loaded into Cu-BTC and its effect on growth characteristics, plant pigments, biochemical components and bioactive components in grains such as avenantheramide C, phenolic acids, vitamins, and yield parameter are as presented in this study.

#### Effect of Cu-BTC, and oats extracts on growth parameters of oat

**Fig. 4:**
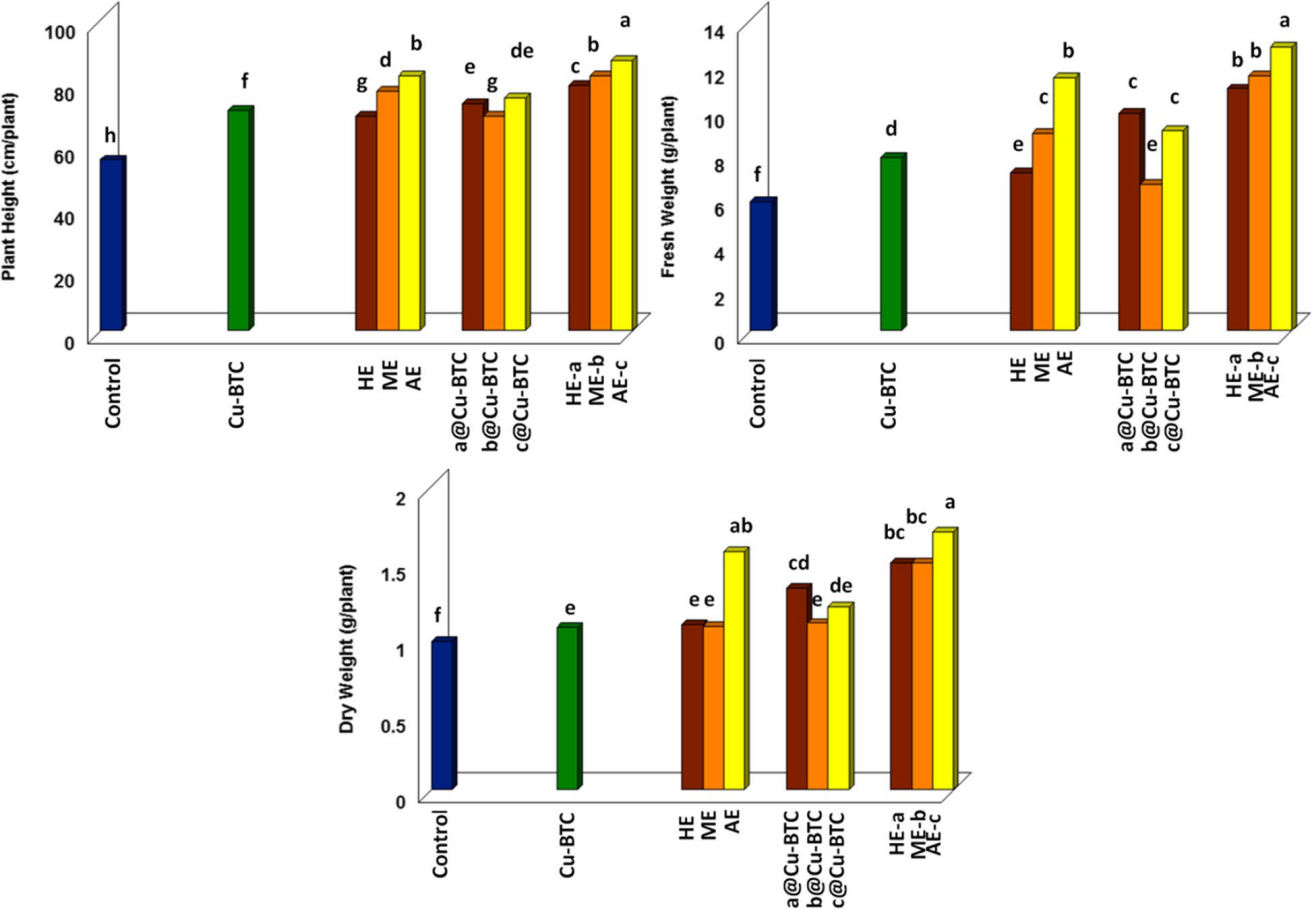
Effect of Cu-BTC, and oat extracts on growth parameter (plant height, fresh and dry weight) after 75 days from sowing under calcareous soil conditions

Figure 4 showed a positive effect for all treatments on all growth parameters. The best treatment that maximizes all growth parameters is oat extracts after Cu-BTC. The data showed a gradual increase in plant height, fresh and dry weight of oat plants sprayed with AE-c followed by ME-b and then HE-a. Data was appeared increase in plant height at rate 1.58, 1.49 and 1.43 fold more than the control as well as, remarkable increase at rate 2.2, 1.98 and 1.88 fold for fresh weight, likewise, data showed increase in dry weight at rate 1.73, 1.53 and 1.53 fold comparison between untreated plants after 75 days from sowing for AE-c, ME-b and HE-a extracts respectively. In the same direction, leaf spraying with AE, ME, and HE resulted in an enhancement of 1.49, 1.4, and 1.25 times for plant height, 1.96, 1.53, and 1.22 times for fresh weight, and 1.6, 1.1, and 1.11 times for dry weight more than the control respectively. Data was appeared slightly increase in plant growth after treated with a@Cu-BTC and c@Cu-BTC about 1.26 and 1.15 times for fresh weight and 1.24 and 1.13 times for dry weight comparison with Cu-BTC respectively.

#### Effect of Cu-BTC, and oats extracts on photosynthetic pigments of oat

**Fig. 5:**
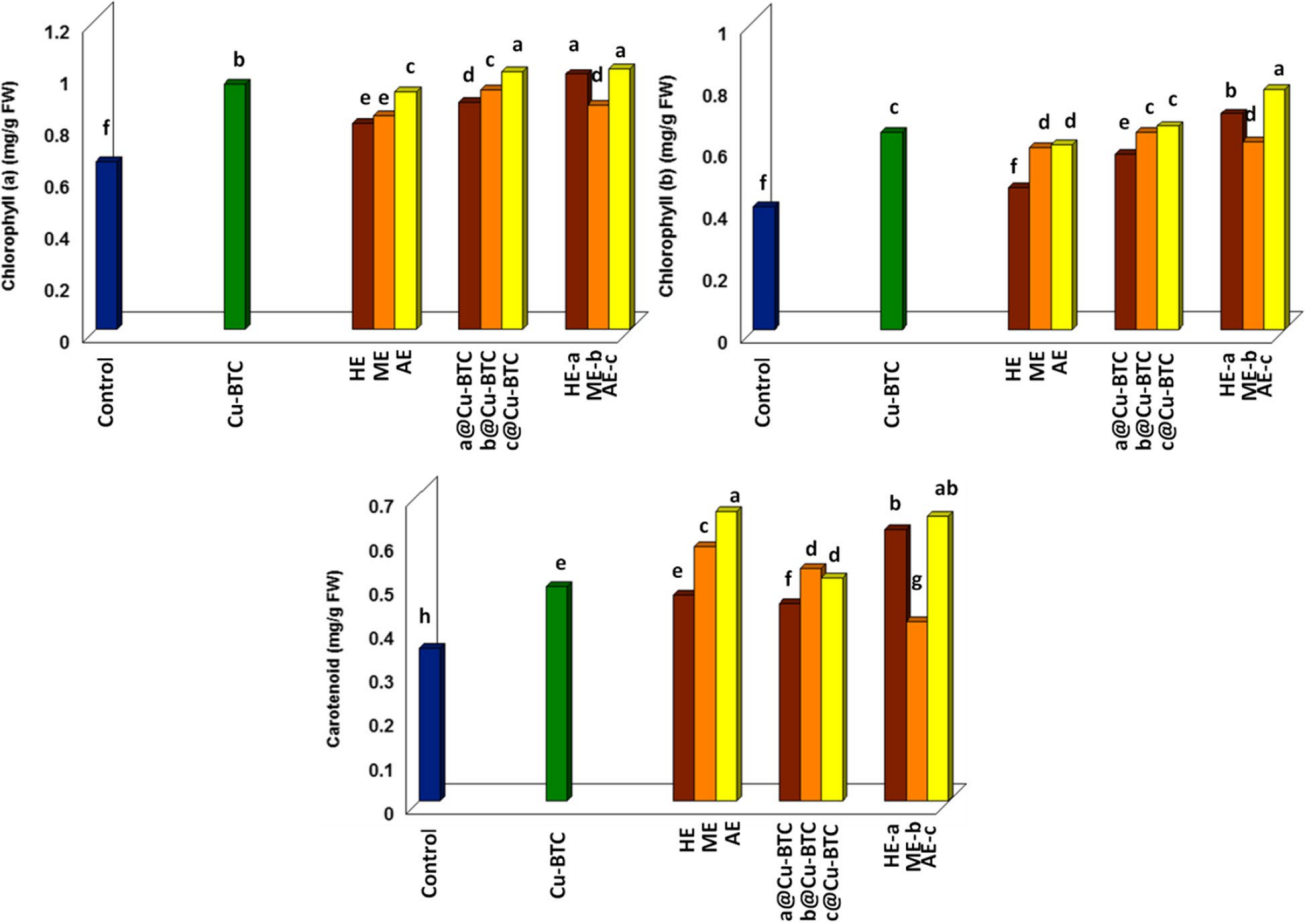
Effect of Cu-BTC, and oat extracts on photosynthetic pigments (chlorophyll a, chlorophyll b, and carotenoid) after 75 days from sowing under calcareous soil conditions

Figure 5 showed spray treatments with different oat extracts (HE, ME, AE, HE-a, ME-b and AE-c) caused enhanced in chlorophyll a, b and carotenoid compared to untreated plants. Where's, data showed that the enhancement that occurred after spraying oat plants with HE, ME, AE, HE-a, ME-b and AE-c was at rates of 1.23, 1.28, 1.42, 1.52, 1.34 and 1.55 times for chlorophyll a, 1.15, 1.47, 1.5, 1.75, 1.52 and 1.95 times for chlorophyll b and 1.34, 1.66, 1.83, 1.77, 1.17 and 1.86 times for carotenoid more than the control respectively. As well as foliar application with c@Cu-BTC let to slightly increase in chlorophyll a, b and carotenoid compared with Cu-BTC, but foliar application with a@Cu-BTC and b@Cu-BTC was caused slightly decline in photosynthetic pigments less than Cu-BTC.

#### Effect of Cu-BTC, and oats extracts on biochemical component of oat

Table 2 exhibited the biochemical components in cultivated oats as affected by foliar treatments of oats extracts and oat extracts loading in Cu-BTC such as protein percent, water-soluble carbohydrates, and free phenolic content. The obtained results in the same table and figure showed that percentage of total protein in oats leaves was moderate developed when HE, ME, AE, HE-a, ME-b and AE-c extracts were applied twice on oats leaves at rates 1.28, 1.03, 1.43, 1.26, 1.18 and 1.56 fold compared with untreated plant respectively. On the other hand foliar sprayed with c@Cu-BTC extract caused slightly decline in protein percentage at rate 8.3% less than Cu-BTC treatment. In this respect treatment by a@Cu-BTC and b@Cu-BTC extracts on oats leaves twice were recorded a good behavior for protein percentage compared with Cu-BTC and control treatments. Also, Table 2 showed that the level of water-soluble carbohydrate was higher when Cu-BTC and all oat extracts at three forms which applied on oats leaves twice time compared with untreated plants. In this regard, water-soluble carbohydrates in oats leaves were enhanced after treatment with HE, ME, AE, HE-a, ME-b and AE-c about 28.3, 11, 4.5, 117, 53.1 and 25.4% more than the stressed plants respectively. Opposite direct, treatments with c@Cu-BTC showed non-significant effects on water soluble carbohydrates. Additionally, foliar applications with a@Cu-BTC and b@Cu-BTC led to markedly decrease in water-soluble carbohydrates content in oat leaves at ratio 23.7 and 29.6% less than Cu-BTC application respectively. In the same table and figure data proved that, remarkable increase in non-enzymetic antioxidant e.g. free phenolic activity was noticed after treatment with Cu-BTC and three oats extracts at all forms compared with control. The best treatment which enhanced free phenolic compounds accumulation in detached leaves of oat was noticeable with AE-c when sprayed twice at ratio 24.6 and 69.6% more than Cu-BTC and control respectively. Likewise, foliar applications with HE, ME, AE, HE-a and ME-b were caused high production of free phenolic at rates 50.6, 56.9, 47.8, 64.5 and 51.9% more than the control respectively.

**Table 2:** Biochemical constituents in dry oat leaves as affected by Cu-BTC treatments and oat extracts after 75 days from sowing under calcareous soil conditions

**Treatments**	Biochemical constituents*		
	**Total protein (g/100 g)**	**Water soluble carbohydrates (mg/g DW)**	**Free phenolic compounds (mg/g DW)**
**Control**	16.89 ± 0.44 e	51.75 ± 0.78 g	9.53 ± 0.14 e
**Cu-BTC**	21.02 ± 0.41 c	93.88 ± 1.81 b	12.97 ± 0.06 d
**HE**	21.71 ± 0.36 c	66.38 ± 1.61 e	14.35 ± 0.22 bc
**ME**	17.47 ± 0.32 e	57.44 ± 0.52 f	14.95 ± 0.05 b
**AE**	24.14 ± 0.28 b	54.07 ± 0.78 fg	14.09 ± 0.12 c
**a@Cu-BTC**	23.99 ± 0.18 b	71.62 ± 1.21 d	14.83 ± 0.13 b
**b@Cu-BTC**	25.08 ± 0.15 ab	66.04 ± 1.41 e	13.91 ± 0.08 c
**c@Cu-BTC**	19.28 ± 0.23 d	95.44 ± 1.21 b	14.31 ± 0.27 bc
**HE-a**	21.27 ± 0.47 c	112.32 ± 1.41 a	15.68 ± 0.18 b
**ME-b**	19.91 ± 0.55 d	79.23 ± 0.33 c	14.48 ± 0.12 c
**AE-c**	26.29 ± 0.29 a	64.87 ± 0.62 e	16.16 ± 0.16 a

*Values followed by the same letter in columns are not different at *p* < 0.05 by Duncan’s multiple range tests. Data are mean of 3 replicates ± Standard error. *DW* Dry weight

#### Effect of Cu-BTC, and oats extracts on oat yield parameter.

Table 3 demonstrated that all treatments caused moderate increase in oat heights after 175 days from sowing compared with unsprayed plants, in the similar trend the HE, ME, AE, HE-a, ME-b and AE-c treatments led to significantly increase in oat heights about 1.14, 1.15, 1.19, 1.24, 1.24 and 1.16 times more than stressed plants respectively. On the other hand, it was found that the spraying treatments using a@Cu-BTC, b@Cu-BTC and c@Cu-BTC did not have any significant effect on oat heights compared to Cu-BTC treatment.

**Table 3:** Plant height and yield of oat (grains and straw) affected by Cu-BTC treatments and oat extracts 175 days after sowing under calcareous soil conditions

**Treatments**	Yield components*		
	**Plant height** **(cm/plant)**	**Grains yield** **(ton/ha)**	**Straw yield** **(ton/ha)**
**Control**	90.21 ± 1.21 c	1.06 ± 0.07 e	1.81 ± 0.05 e
**Cu-BTC**	103.23 ± 1.71 b	1.55 ± 0.04 cd	2.07 ± 0.07 d
**HE**	103.11 ± 2.01 b	1.58 ± 0.08 cd	2.45 ± 0.23 c
**ME**	104.21 ± 0.71 b	1.79 ± 0.02 b	2.87 ± 0.05 b
**AE**	107.32 ± 3.41 ab	1.62 ± 0.07 c	3.12 ± 0.04 a
**a@Cu-BTC**	107.44 ± 1.41 ab	1.62 ± 0.02 c	2.49 ± 0.09 c
**b@Cu-BTC**	107.12 ± 1.22 ab	1.69 ± 0.06 c	2.85 ± 0.04 b
**c@Cu-BTC**	103.12 ± 1.23 b	1.68 ± 0.32 c	2.82 ± 0.26 b
**HE-a**	112.11 ± 3.15 a	1.88 ± 0.01 b	2.94 ± 0.06 ab
**ME-b**	112.21 ± 0.34 a	2.01 ± 0.12 ab	3.00 ± 0.16 a
**AE-c**	104.12 ± 3.52 b	2.19 ± 0.07 a	2.96 ± 0.08 ab

^*^Values followed by the same letter in columns are not different at *p* < 0.05 by Duncan’s multiple range tests. Data are mean of 3 replicates ± Standard error

Table 3 showed that foliar application of AE-c, ME-b and HE-a more effective on grains yield than other applications, these treatments created enhancement in grain yield at rates 2.07, 1.89 and 1.77 fold, while foliar sprayed with AE, ME and HE extracts on oat leaves led to significantly increase in grains yield about 1.53, 1.69 and 1.49 times more than the control, respectively. Likewise foliar treatment with Cu-BTC created moderate increase in grains yield at rate 1.46 fold more than stressed plants. But foliar treatments with a@Cu-BTC, b@Cu-BTC and c@Cu-BTC caused slightly enhanced in grains yield compared to Cu-BTC treatment.

Also, all applications caused developed in straw yield compared with control. In the light of this, data showed that the largest amount of straw yield was for AE and ME-b, followed by AE-c, HE-a and ME then HE at rates 1.72, 1.66, 1.64, 1.62, 1.58 and 1.35 compared to untreated plants respectively. Foliar application with Cu-BTC led to mild enhanced in straw yield compared with control; while leaves sprayed with a@Cu-BTC, b@Cu-BTC and c@Cu-BTC were appeared moderate increase in straw yield compared with Cu-BTC treatment.

#### Effect of Cu-BTC, and oats extracts on bioactive components in oat grains

Data in Table 4 and Figure (S7, S8 and S9) investigated the free phenolic compounds and avenantheramide C were separated by using HPLC chromatography system (Agilent 1100) results showed that the processed oats with Cu-BTC, HE, ME, AE, a@Cu-BTC, b@Cu-BTC, c@Cu-BTC, HE-a, ME-b and AE-c were contained 13 free phenolic compounds in addition to avenantheramide C. All treatments with Cu-BTC, HE, ME, AE, a@Cu-BTC, b@Cu-BTC, c@Cu-BTC, HE-a, ME-b and AE-c showed marked improvements in avenantheramide C about 1.59, 4.03, 3.58, 4.35, 1.44, 2.92, 3.58, 3.32, 1.89 and 7.57 times more than the control respectively. Likewise all sprayed applications were appeared enhancement in free phenolic acids content except b@Cu-BTC and Cu-BTC showed milled reduced in free phenolic content compared with untreated plants. Cu-BTC foliar sprayed clarified that ellagic acid, cinnamic acid and caffeic acid were appeared most abundant at rate 30.8, 27.3 and 18.05% then ferulic acid and salicylic acid at rate 13.81 and 10.04% respectively. HE foliar spray resulted to higher amount in cinnamic acid, protocatechulic acid, P-OH benzoic acid, and caffeic acid about 29.26, 24.29, 20.45 and 15.09% then catechol and chlorogenic acid were less amount at 9.2 and 1.7% respectively. ME application showed that gallic acid, syringenic acid and cinnamic acid were higher amount at 47.6, 19.27 and 12.64% and catechol, ellagic acid, ferulic acid and chlorogenic acid were fewer amounts at 9.86, 6.8, 2.68 and 1.15% respectively. AE treatment appeared that big amount in ellagic acid, cinnamic acid, gallic acid and caffeic acid were formed about range 23.14, 20.43, 17.42 and 14.44% followed by ferulic acid, syringenic acid and salicylic acid about 11.18, 9.8 and 3.35% respectively. a@Cu-BTC treatment gave protocatechulic, ferulic acid and salicylic acid were the higher concentration at 28.13, 22.66 and 21.45% then pyrogallol, cinnamic acid, *P*-hydroxybenzoic acid and caffeic acid at 15.26, 7.5, 3.43 and 1.56% respectively. b@Cu-BTC sprayed led to syringenic acid, *P*-hydroxybenzoic acid and gallic acid were higher quantity at rang 34.95, 27.03, and 21.12% then caffeic acid and cinnamic acid were lower quantity with range 16.77 and 0.28% respectively. c@Cu-BTC foliar application showed that *O*-coumaric acid, salicylic acid, cinnamic acid were maximized formed at rate 24.16, 17.16 and 15.79% then ellagic acid, ferulic acid, gallic acid and syringenic acid were minimized at 13.13, 13.0, 8.56 and 8.18% respectively. Also, HE-a treatment was performed resulted in the formation of chlorogenic acid, *P*-hydroxybenzoic acid, ferulic acid, gallic acid and *O*-coumaric acid with ranges between 27.02,19.49, 17.71, 17.27 and 13.8% but salicylic acid, ellagic acid, caffeic acid and protocatechulic acid were lower formed at 1.8, 1.59, 1.02 and 0.3% respectively. ME-b treatment gives rise to ferulic acid and caffeic were amplified quantity about 36.68 and 14.98% followed by gallic acid, chlorogenic acid, cinnamic acid and protocatechulic at 13.53, 12.81, 12.15 and 9.85% respectively. AE-c treatment showed the ellagic acid and ferulic acid were the most abundant at 25.18 and 20.42% followed by catechol and syringenic acid at 13.25 and 12.6% then gallic acid, cinnamic acid and caffeic acid at 11.14, 11.09 and 6.28% respectively.

**Table 4:** Phenolic compounds and avenanthramide-C content in oat grains (µg/g) affected by Cu-BTC treatments and oat extracts under calcareous soil conditions

**Free phenolic compounds**	**Foliar applications**										
	**Control**	**Cu-BTC**	**HE**	**ME**	**AE**	**a@Cu-BTC**	**b@Cu-BTC**	**c@Cu-BTC**	**HE-a**	**ME-b**	**AE-c**
**Catechol**	nd	nd	0.748	0.774	nd	nd	nd	nd	nd	nd	1.828
**Caffeiec acid**	nd	1.132	1.226	nd	1.844	0.112	0.932	nd	0.088	1.122	0.866
**Ferulic acid**	3.066	0.866	nd	0.21	1.428	1.624	nd	1.234	1.532	2.748	2.816
***O*-Coumaric acid**	nd	nd	nd	nd	nd	nd	nd	2.292	1.194	nd	nd
**Gallic acid**	0.836	nd	nd	3.736	2.258	nd	1.174	0.812	1.494	1.014	1.536
**Chlorogenic acid**	0.916	nd	0.138	0.09	nd	nd	nd	nd	2.338	0.96	nd
**Syringenic acid**	nd	nd	nd	1.512	1.25	nd	1.934	0.776	nd	nd	1.738
**P-OH benzoic acid**	nd	nd	1.662	nd	nd	0.246	1.502	nd	1.686	nd	nd
**Cinnamic acid**	nd	1.712	2.378	0.992	2.61	0.538	0.0156	1.498	nd	0.91	1.53
**Salicylic acid**	1.196	0.63	nd	nd	0.428	1.538	nd	1.628	0.156	nd	nd
**Ellagic acid**	nd	1.932	nd	0.534	2.956	nd	nd	1.246	0.138	nd	3.472
**Pyrogallol**	nd	nd	nd	nd	nd	1.094	nd	nd	nd	nd	nd
**Protocatechulic**	0.732	nd	1.974	nd	nd	2.016	nd	nd	0.026	0.738	nd
**Cumulative free phenolics**	**6.746**	**6.272**	**8.126**	**7.848**	**12.774**	**7.168**	**5.5576**	**9.486**	**8.652**	**7.492**	**13.786**
**Avenantheramide C**	14.262	22.7	57.47	51.094	62.112	20.564	41.634	51.042	47.29	27.038	107.95

*nd* Not detectabl

^a^Where; phenolic acids in the table were arranged (Ascending) according to the retention time of phenolic acids which separated from C_18_ column of HPLC apparatus

Table 5 and Figures (S10 and S11) showed that fat-soluble vitamins are present in oat grains. The vitamins content in grains are vitamin α-tocopherol (E) and phylloquinone (K). Foliar spraying with AE-c was superior on all foliar applications in vitamins α-tocopherol (E) and K accumulation in oat grains at rate 13.05 and 9.16 mg/g DW respectively. Also, foliar application with a@Cu-BTC was appeared increase in vitamin α-tocopherol aggregation at range 10.1 mg/g DW noticeably but vitamin (K) not detect. In this regard, all foliar spraying with ME-b, HE, c@Cu-BTC, ME, AE, HE-a and b@Cu-BTC were led to excess in over production of vitamin E in oat grains about 1.7, 1.57, 1.5, 1.42, 1.4, 1.29 and 1.1 times more than the control respectively. While Cu-BTC treatments showed that decrease in vitamin E level about 32% less than the control. In the same direction, all treatments caused enhancement of vitamin K in oat grains except HE treatment. The results showed an increase in vitamin K concentration by 7.25, 4.7, 4.6, 3.1, 2.8, 2.1 and 1.9 times when foliar spraying with treatments HE-a, b@Cu-BTC, Cu-BTC, ME-b, AE, c@Cu-BTC and ME compared to control respectively.

**Table 5:** Fat-soluble vitamins contained in oats grains (mg/g) after spraying with Cu-BTC and oat extracted under calcareous soil conditions

Treatments	Vitamins compounds (mg/g DW)^a^	
	*α* Tocopherol (vitamin E)	*Vitamin K*
**Control**	5.42	1.12
**Cu-BTC**	3.66	5.14
**HE**	8.47	0.98
**ME**	7.69	2.14
**AE**	7.55	3.11
**a@Cu-BTC**	10.12	nd
**b@Cu-BTC**	5.69	5.26
**c@Cu-BTC**	7.88	2.36
**HE-a**	6.98	8.12
**ME-b**	9.16	3.44
**AE-c**	13.05	9.16

*nd* Not detectable

^a^Where; vitamins were separated from C_18_ column of HPLC apparatus

### Anti-cancer activity

**Fig. 6:**
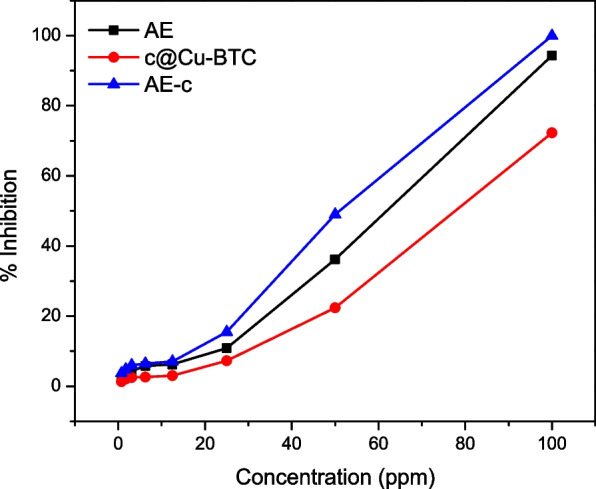
Dose response curve of target component on breast cancer cell line MCF 7

AE, c@Cu-BTC and AE-c components exhibits excellent anticancer activity against tested cell lines MCF 7. The MTT test was performed to analyze the prepared component’s anticancer activity. The result shows that AE, c@Cu-BTC and AE-c have 94.3, 72.3, and 100% (Fig. 6). These findings clearly suggest that aqueous extraction from oats possess anti-tumor activity potential, and more research is needed to validate the current findings. The anticancer effects of oat grain was related to vitamin K and avenanthramide group [38].

## Discussion

Oat plant growth factor measurements as shown in Fig. 4 have the same trend as found in the work done on *Phaseolus vulgaris* [24]. Salicylic acid can inhibit shoot growth while promoting root formation and, in some species, flowering [39]. The increase of plant heights may be related to: 1) oat extracts containing a large number of important constituents and phytochemicals including proteins [40]. Protein metabolism contributes to the synthesis of auxin, salicylic acid and IAA which effects on plant growth and controlled in a range of processes, such as maintenance of apical dominance, shoot elongation, and root initiation [41, 42]; 2) the main components of polyunsaturated fatty acid in oat bran oil is linoleic acid [43]. Plants synthesize jasmonic acid from linoleic acid which has effects on seed germination, fertility and root growth [44]; 3)MOFs was release natural bioactive molecules of oat extracts and natural molecules less effective have kept inside MOFs structure [27, 45]. Additionally**,** increase in biomass accumulation may be due to: 1) increase in water content and nutrient solution in plants [24]; 2) whole oat groats contain high levels of nutrients such as soluble fiber, carbohydrates, proteins, unsaturated fatty acids, vitamins, minerals and other phytochemicals [40]; 3) protein is a precursor of enzyme synthesis which contribution in cytokinins and gibberellins biosynthesis have promote cell division [46]; 4) high level of phenolic compounds in oat extracts and play key role in cell division consequently plant growth and development including seed germination, biomass accumulation, and improved plant metabolism [47].

Figure 5 showed strong enhancement in photosynthetic pigments return to aqueous extract AE-c and AE treatments in fresh oats leaves. The obtained results are confirmed with previous work [24]. The positive effect of aqueous extract on chlorophyll content may be due to: 1) oat aqueous extract contained many bioactive compounds such as non-enzymatic antioxidant e.g. phenols, flavonoids and vitamins (A,B and E) [40, 48, 49]; 2) the existing mechanism behind these flavonoids is iron chelating process and it also deals with proteins phosphorylation [50]; 3) the importance of iron in relation to the plant lies in the fact that it enter into many physiological processes e.g. biosynthesis of cytochromes, chlorophyll, the electron transport system and building of iron sulfur clusters [51, 52].

Table 2 were supported by previous work which reported that the protein content of oat groat ranged from 12–24% [53], also the obtained data is consisted with the work done on treatment of *Phaseolus vulgaris* with Fe-MOFs-EDTA, they found that protein content was increased progressively when treated with material [24]. More fitting to our data the work done on oat bran found that protein was 13–20% [10]. High accumulation of protein in detached oat leaves may be return to during extractions and preparation of protein in oat grain, a degradation of amino acids which a precursor to de novo synthesis of protein also amino acids contribution in biosynthesis of enzymes and hormones to help increase of protein biosynthesis [54]. Table 2 were in complete harmony with previous work [55]. The development in soluble carbohydrate may be due to: 1) high level of carbohydrate in *Avena sativa* L. grains ranged 60% [10, 40]; 2)the phenomenal properties of MOFs materials including oxalate which a dicarboxylic acid during it metabolism plants use oxalate as a carbon source to satisfy their energy metabolism requirements, resulting in the formation of carbonates, which is a metabolic process known as the oxalate-carbonate pathway [56, 57]. Also, these results were strongly supported by previous work on *Avena sativa* L., they observed that phenols qualitative were abundant in ethanol and n-butanol fraction [58]. Likewise, the co-solvent had a positive effect on phenols content in oat plants [59]. High level of phenolic content may be due to: 1) high tyrosinase enzyme inhibition and accumulation of tyrosine in ethanol fraction subsequently n-hexane fraction of *Avena sativa*); 2) high level of protein in *Avena sativa* L. which converted to aromatic amino acids like phenylalanine, tryptophane and tyrosine as a precursor of phenolic biosynthesis [40]. Phenolic compounds were biosynthesized through the secondary metabolite in plants. Phenolic compounds participate in the biosynthesis of antioxidants, structural polymers (lignin), and attractants (flavonoids and carotenoids). Anti-aging, anti-inflammatory, antioxidant, and anti-proliferative activities of phenolic compounds are critical in defence responses. As a result, it is advantageous to consume plant foods with high antioxidant compound content, which will reduce the incidence of certain chronic diseases, such as diabetes, cancer, and cardiovascular disease [60].

Table 3 were supported by previous work [61], they reported that aqueous root extract of oat was observed up to 2% stimulatory effect on elongation of mung bean plant. The ameliorative effect of oat extracts may be related to: 1) highly competitive of oat extracts often exhibit early vigorous growth, high tillering capacity, increased plants height [62]. Furthermore, oat extracts excellent nutritional value, with the more effective on yielding better nutrition for plants [40]. The MOFs can be contact with harmful chemical compounds that negatively affect plant growth [63]. Too, These development in grains and straw yield may be returned to: 1) MOFs excellent of crystalline materials that have adsorption of injurious gases at surfaces such as CO and NH_3_ reflected on general health of plants [64]; 2) ameliorative effects of *Avena sativa* extracts have bio-stimulant products as well as stimulation of plant secondary metabolism [65, 66].

Table 4 were in complete harmony with pervious work [67], they reported that oat extract containing on eight kind of avenanthramides; and the work in eight Finnish cultivars of *Avena sativa* and found the avenanthramides levels ranged from 26.7 to 185 mg/kg [68]. The amount of avenanthramides in oat grains ranges between 2 mg/kg-53 mg/kg [69]. As well, Table 4 showed a good harmony with previous studies [40, 68] who separated eight types of phenolic compounds from the oat cultivars. *Avena sativa* are containing on p-hydroxybenzoic acid, vanillic acid, avenanthramide (A, K and C),caffiec acid and vanillin [58]. Likewise, syringic acid, p-coumaric acid, protocatechuric acid, gallic acid and ferulic acid was separated from *Avena sativa* seeds [70]. The increment of phenolic acids after treatment with oats extract and MOFs may be due to: 1) oat extracts are containing on the group of phenolic compounds and aromatic amino acids like tryptophan, tyrosine and phenylalanine biosynthesis induces of mono-phenols (simple phenolic acids) such as benzoic acid derivatives (hydroxyl-benzoic acids) and cinnamic acid derivatives (hydroxyl-cinnamic acids) [71]; 2) oat extracts are including on carbon skeletons for many essential compounds such as salicylic acid and quinines [72, 73]; 3) oat extracts are containing on many enzymes contribution in shikimate pathway and it regulate the phenolic acids biosynthesis [71].

Moreover, Table 5 was in complete harmony with study in oat grains, it was found that different kinds of vitamins ( B6 and B12), vitamin A and tocols were located in the plant [74, 75]. The highest values of vitamins in oat grains may be related to: 1) oat extracts contain many aromatic amino acids, such as phenylalanine tryptophan and tyrosine, as well as shikimic acid, which formation chorismate. It is a precursor to the redox-active naphthoquinone ring of phylloquinone (vitamin K) [76]; 2) oat extracts are including specificity substrate to motivate DHNA-CoA activity and phylloquinone(vitamin K) content [77]; 3) MOFs are methylated donors, which contribute to the methylation of the chromanol ring that contributes to the biosynthesis of vitamin E, in addition, oat extracts contain non-polar lipids such as γ-tocopherol, which is a precursor to vitamin E synthesis [78]. The best treatment that gives high grain yield with the most increased anti-cancer activity was AE-c.

## Conclusion

From the previous results we found that, presence of various allelochemicals acts as inducer/inhibitor growth factors. In this study, we were successfully able to separate some allelochemicals compounds from different oat extracts (HE, ME, and AE) using Cu-BTC MOFs, depending on the configuration shape of MOF. HPLC and GC-Mass were used for identification and quantification those compounds. The promising finding is the separation of triacylglycerol (a) from HE, anthraquinone (b) from ME and salicylic acid (c) from AE. The study showed a significant and clear improvement on plants whose young leaves were sprayed with different oat extracts (HE, ME, AE, a@Cu-BTC, b@Cu-BTC, c@Cu-BTC, HE-a, ME-b and AE-c) under calcareous soil conditions. Plants sprayed with HE-a, ME-b, and AE-c showed a high positive effect on oats morphology, and this appeared in the results of growth characteristics, plant pigments and yield components, as well as an improvement in the endogenous chemical components of oats, which is represented by total protein, water-soluble carbohydrates, and phenolics compounds. Moreover, there was a clear enhancement in the content of oat grains for antioxidants in terms of their type and quantity such as the types and quantities of free phenolic acids like ellagic acid, ferulic acid and chlorogenic acid, polyphenol as avenantheramide C, and fat-soluble vitamins such as vitamin E and vitamin K which acts as an anti-cancer. Moreover, a clear and significant positive effect of AE-c on cancer cells was demonstrated in a cell line study. While spraying with whole oat extracts (HE, ME and AE) had a moderate effect on the factors of the previous study. But spraying with Cu-BTC containing on allelochemicals compounds (a, b, and c) showed a minimum effect on the factors of the previous study compared to other extracts and therefore these compounds are considered growth inhibitors. Finally, the use of MOFs in the agricultural application was maximized by absorbing some growth-inhibiting compounds on their surface and increasing the efficacy of various oat extracts in inducing growth and yield improvement under calcareous soil conditions.

## Supplementary Information

**Additional file 1: Supporting information**. **Figure S1.** GC chromatogram for hexan extract of oat grains. **Figure S2.** GC chromatogram for hexan extract of oat grains after path through Cu-BTC. **Figure S3.** HPLC chromatogram for methanol extract of oat grains. **Figure S4.** HPLC chromatogram for methanol extract of oat grains after path through Cu-BTC. **Figure S5.** HPLC chromatogram for water extract of oat grains. **Figure S6.** HPLC chromatogram for water extract of oat grains after path through Cu-BTC. **Figure S7.** Effect of foliar applications (Cu-BTC, HE, ME, and AE) on phenolic acids, and avenantheramide-C content in oat grains under calcareous soil conditions. **Figure S8.** Effect of foliar applications (a@Cu-BTC, b@Cu-BTC, and c@Cu-BTC) on phenolic acids, and avenantheramide-C content in oat grains under calcareous soil conditions. **Figure S9.** Effect of foliar applications (HE-a, ME-b, and AE-c) on phenolic acids, and avenantheramide-C content in oat grains under calcareous soil conditions. **Figure S10.** Effect of foliar applications (Cu-BTC, HE, ME, AE, and a@Cu-BTC) on vitamins E and K, content in oat grains under calcareous soil conditions. **Figure S11.** Effect of foliar applications (b@Cu-BTC, c@Cu-BTC, HE-a, ME-b, and AE-c) on vitamins E and K, content in oat grains under calcareous soil conditions. **Table S1.** Chemical analysis of the experimental soil and underground irrigation water at Maryout station. **Table S2.** The meteorological data at Maryout site.

## Data Availability

The data are available in a Supplementary file.
