# Supplementary material for: Boosting vegetation, biochemical constituents, grain yield and anti-cancer performance of cultivated oat (Avena sativa L) in calcareous soil using oat extracts coated inside nanocarriers
Source: BMC Plant Biol. 2022 Nov 24;22:544. doi: 10.1186/s12870-022-03926-w (PMC9700955; doi:10.1186/s12870-022-03926-w)
Supplement: Supplementary file 1 — Additional file 1: Supporting information. Figure S1. GC chromatogram for hexan extract of oat grains. Figure S2. GC chromatogram for hexan extract of oat grains after path through Cu-BTC. Figure S3. HPLC chromatogram for methanol extract of oat grains. Figure S4. HPLC chromatogram for methanol extract of oat grains after path through Cu-BTC. Figure S5. HPLC chromatogram for water extract of oat grains. Figure S6. HPLC chromatogram for water extract of oat grains after path through Cu-BTC. Figure S7. Effect of foliar applications (Cu-BTC, HE, ME, and AE) on phenolic acids, and avenantheramide-C content in oat grains under calcareous soil conditions. Figure S8. Effect of foliar applications (a@Cu-BTC, b@Cu-BTC, and c@Cu-BTC) on phenolic acids, and avenantheramide-C content in oat grains under calcareous soil conditions. Figure S9. Effect of foliar applications (HE-a, ME-b, and AE-c) on phenolic acids, and avenantheramide-C content in oat grains under calcareous soil conditions. Figure S10. Effect of foliar applications (Cu-BTC, HE, ME, AE, and a@Cu-BTC) on vitamins E and K, content in oat grains under calcareous soil conditions. Figure S11. Effect of foliar applications (b@Cu-BTC, c@Cu-BTC, HE-a, ME-b, and AE-c) on vitamins E and K, content in oat grains under calcareous soil conditions. Table S1. Chemical analysis of the experimental soil and underground irrigation water at Maryout station. Table S2. The meteorological data at Maryout site. [file 12870_2022_3926_MOESM1_ESM.docx]

**Supporting information**

**Material and methods**

- 1. **Growth measurement**

Plant heights, fresh and dry weight of the whole plants were expressed as centimeter (cm) and grams (g), respectively. Dry weights were recorded after drying the plant samples in oven at 60 °C until constant weight.

- 1. **Photosynthetic pigment measurement**

Chlorophyll a, b and carotenoid were extracted and estimated in fresh leaves according to (Horwitz et al., 1970)

- 1. **Total water soluble carbohydrate content**

Plant samples of 0.2 g oven-dried leaves were extracted twice in 20 ml of aqueous (80% v/v) boiling ethanol for three hours under reflux in water bath and the fractions were collected, cleared by filtering through Whatman no. 42 paper (Kerepesi et al., 1996)**.** Filtrates were completed the volume to 50 ml with 80% ethanol and total water soluble carbohydrate determined based on the phenol-sulfuric acid method (Dubois et al., 1956)**.** The basic principle of this method is that carbohydrates, when dehydrated by reaction with concentrated sulfuric acid, produce furfural derivatives. Further reaction between furfural derivatives and phenol develops detectible color. Involved adding 1 ml of 5% phenol solution to 1 ml plant extract and 5 ml of concentrated sulfuric acid is added rabidly, they are vortexes for 30 sec and reading the absorbance at 490 nm after 20 min. sucrose was used as standard.

- 1. **Free phenolic compounds estimated**

Dried leaves powder was weighted 0.5 g extracted with aqueous/methanol (1:1 v/v) for extraction and hydrolysis according to (Stratil et al., 2006), supernatant were collected and completed the volume to 50 ml with 50% methanol. Free phenolic compounds were quantification by the Folin-Ciocalteu method, based on the reduction of a phosphowolframate-phosphomolybdate complex by phenolics to blue reaction products (Vinson et al., 1998; Vinson et al., 2001)**.** The samples were read at 760 nm by using spectrophotometer and gallic acid as standards.

- 1. **Total protein content**

Nitrogen protein was estimated by multiplying the total
nitrogen by 6.25 (Aoac, 1990)**.** Total nitrogen was determined by Kjeldah procedure. Digestion step, oven dried plant sample 0.25 g was weighed and subjected to a high temperature digestion with concentrated H_2_SO_4_ and catalysts H_2_O_2_ to convert organic and inorganic forms of N to ammonium. Titration step, ammonium in the digest is determined by acidimetric titration following alkaline distillation of ammonia **(A.O.A.C., 1970)** and calculations were done according to (Magomya et al., 2014)**.**

- 1. **Extraction of Free phenolic acids and Avenanthramide-C**

Free phenolic acids were extracted as described by Multari et al. (2018)as follows: oat grains samples were grinded (approximately 0.1 g) were suspended in HCl (3 mL; 0.2 M) and extracted into EtOAc (6 mL), and the layers were separated by centrifugation (5 min; 8000 rpm; 18 °C). The extraction was repeated twice, and the EtOAc extracts were combined and left to stand over sodium sulfate (anhydrous) and then filtered. The solvent was removed under reduced pressure at a temperature not exceeding 40 °C.

Avenanthramide-C was extracted by adapting the method from Bryngelsson et al., 2002. Briefly, milled oat samples (5.0 g) were extracted twice with 80% methanol (35 mL) for 30 min at room temperature using a magnetic stirrer. Then, samples were centrifuged (10 min; 6000 rpm; 18 °C), and the supernatants were dried under reduced pressure at a temperature not exceeding 40 °C. Phenolic and Avenanthramide extracts were dissolved in methanol (1 mL), filtered through PTFE membrane filters, and analyzed by HPLC.

- 1. **Extraction of vitamins**

Vitamins K and E have been extracted individually from oat grains using a mixture of propan-2-ol–hexane and ethanol alcohol or hexane respectively (Booth et al., 1994; Labadie and Boufford, 1988). The extraction was preferred in order to extract vitamins K and E from oat plant Conventional Soxhlet extraction method was applied to samples prior to the HPLC analysis. A 20 g oat grain powder was refluxed with 400 mL of organic solvent for 8 h at the boiling temperature of ethanol by using Soxhlet apparatus. Five parallel extractions were carried for repetition analysis. The extracts were filtered and concentrated by using a rotary evaporator until dryness. Approximately, 1 g of concentrated sample was obtained from each extraction system. In the second step, the obtained extracts were re-dissolved in methanol. 100 mg of extract was weighed and 2.5 mL of solvent was added onto the extracts. Then, the samples were mixed vigorously by a vortex for 10 minutes. Finally, the samples were filtered by using 0.45 µm membrane filter and transferred to HPLC vials according to (Ulusoy et al., 2017).

**
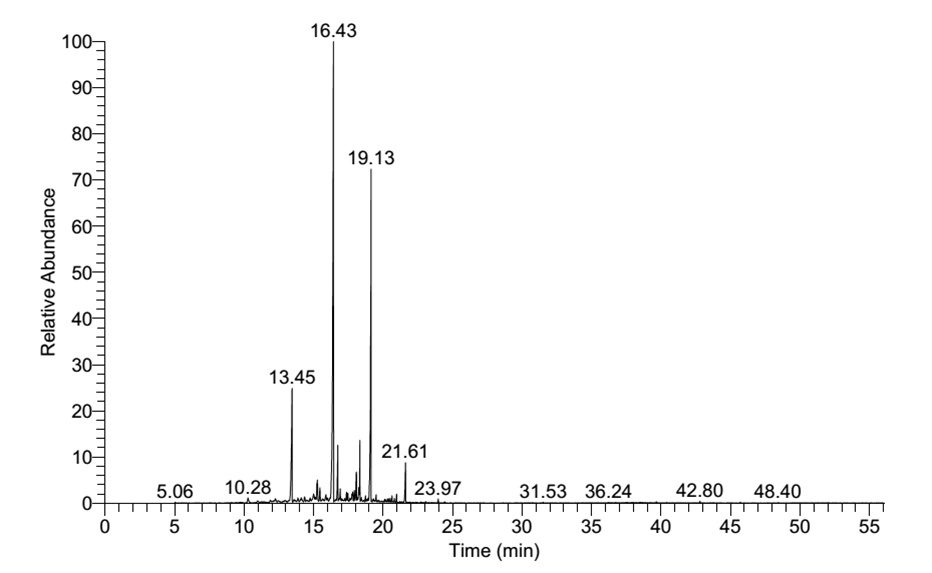
**

**Figure S1 GC chromatogram for hexan extract of oat grains**

**
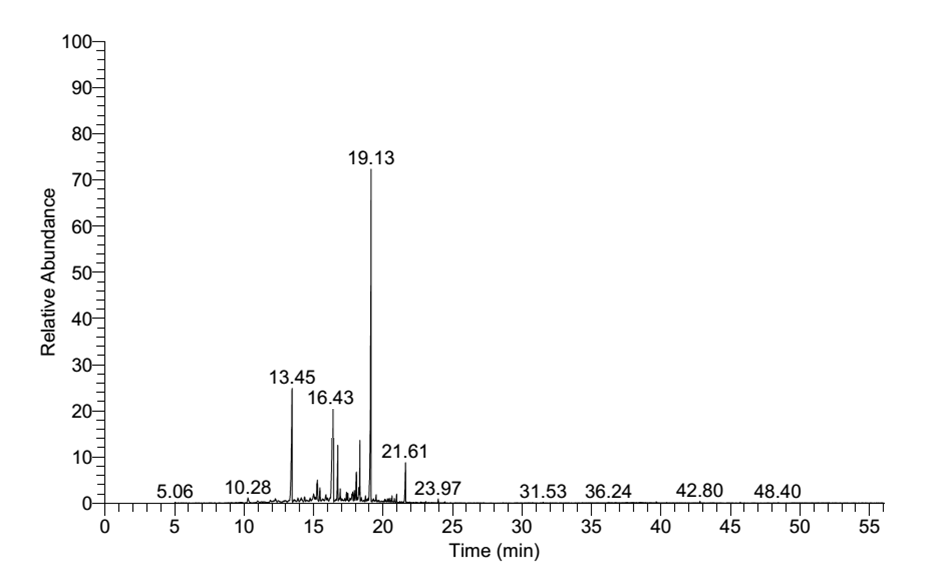
**

**Figure S2 GC chromatogram for hexan extract of oat grains after path through Cu-BTC.**

**
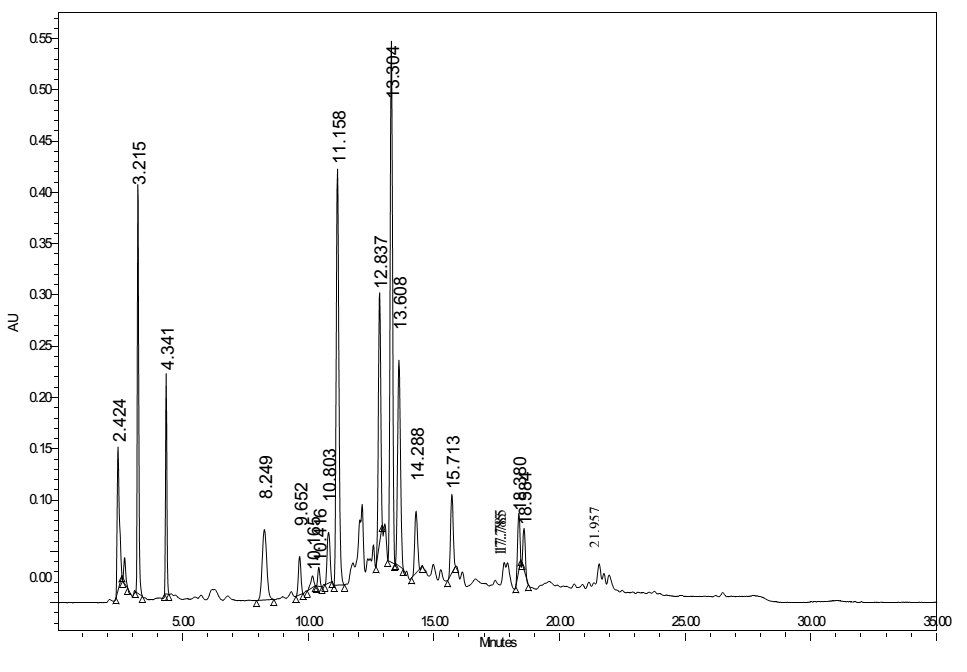
**

**Figure S3 HPLC chromatogram for methanol extract of oat grains**

**
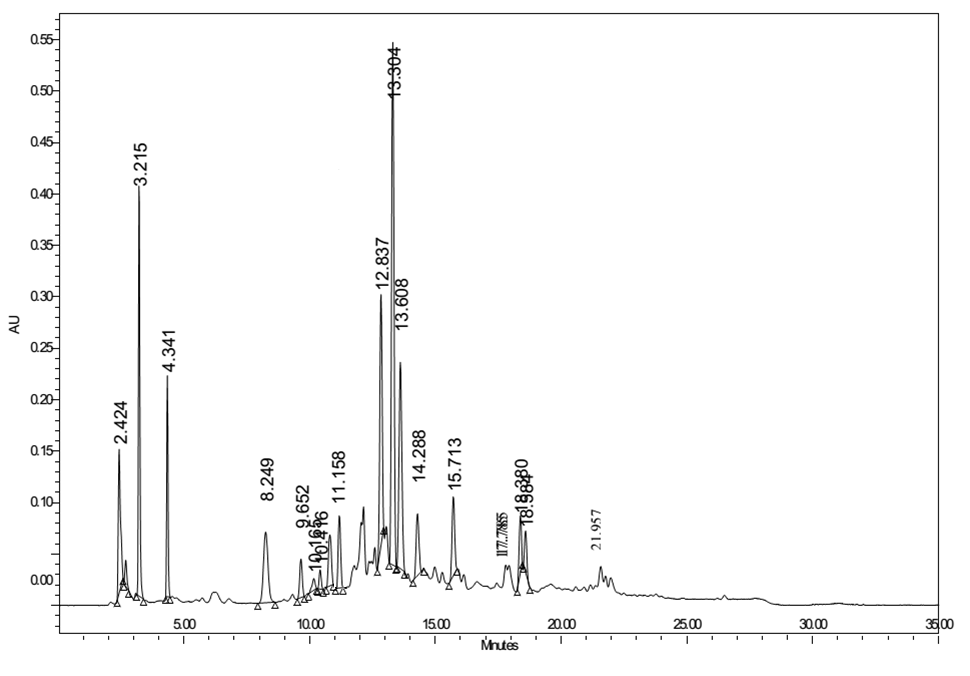
**

**Figure S4 HPLC chromatogram for methanol extract of oat grains after path through Cu-BTC.**

**
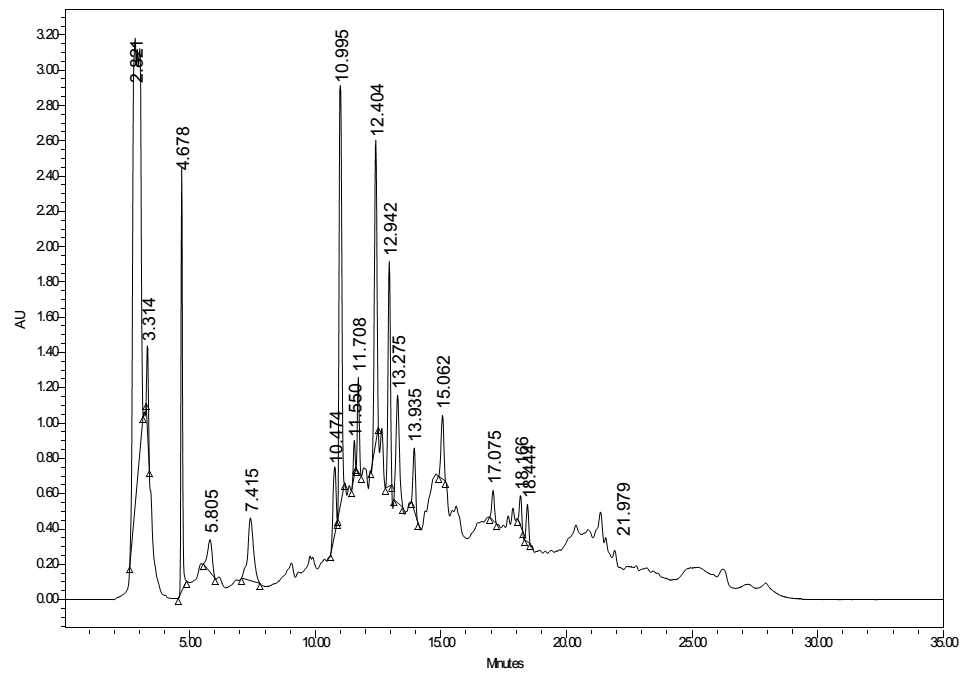
**

**Figure S5 HPLC chromatogram for water extract of oat grains**

**
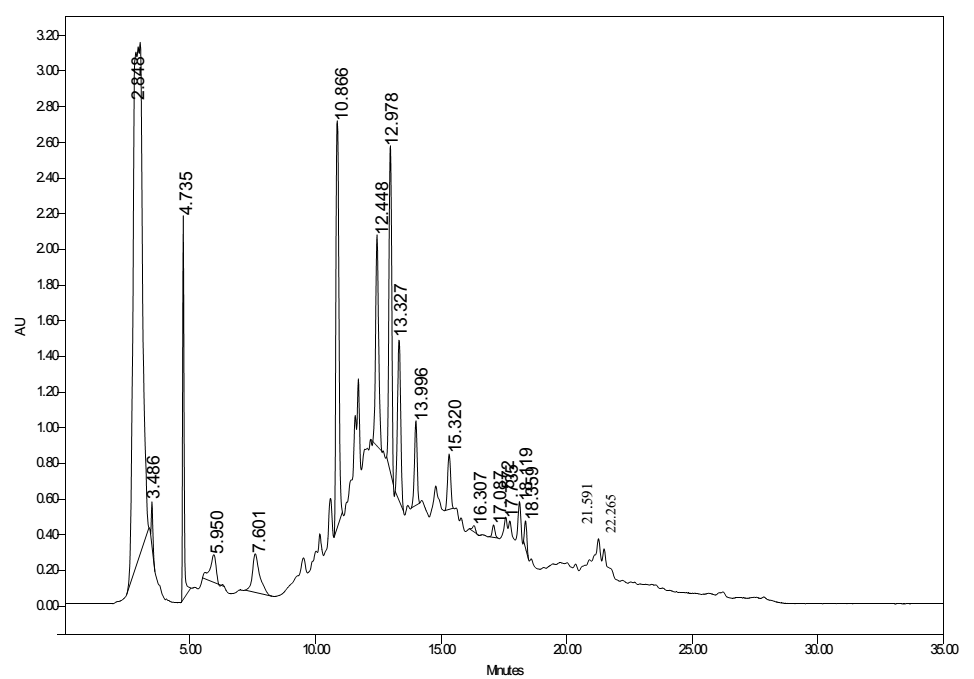
**

**Figure S6 HPLC chromatogram for water extract of oat grains after path through Cu-BTC.**

**Figure S7.** Effect of foliar applications (Cu-BTC, HE, ME, and AE) on phenolic acids, and avenantheramide-C content in oat grains under calcareous soil conditions

| Treatments | Phenolic acids by HPLC | Avenantheramide-C |
| --- | --- | --- |
| Control | 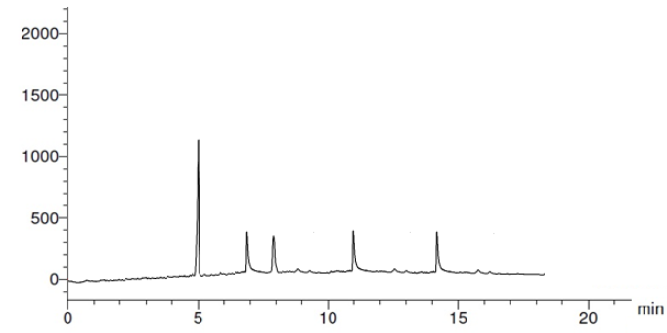 | 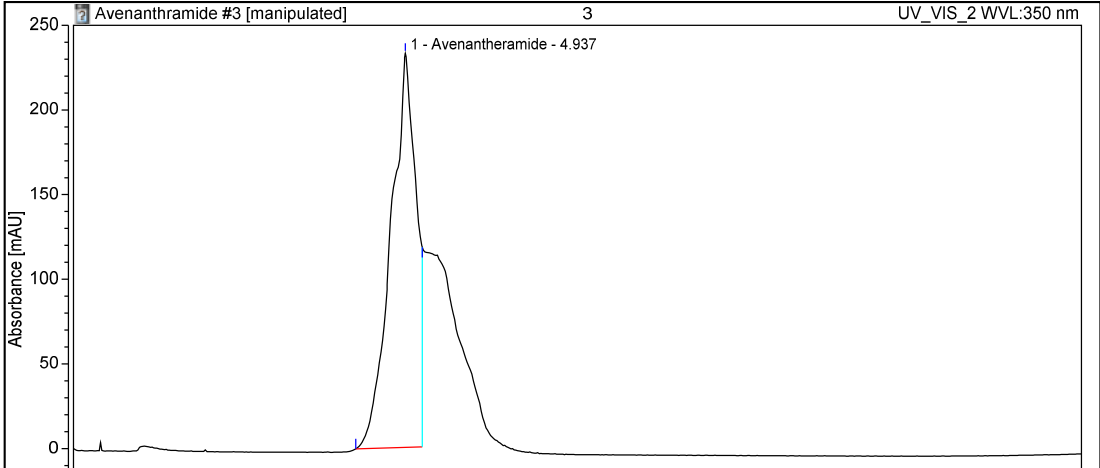 |
| Cu-BTC | 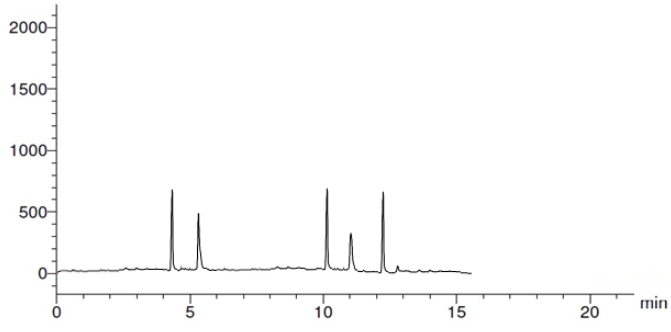 | 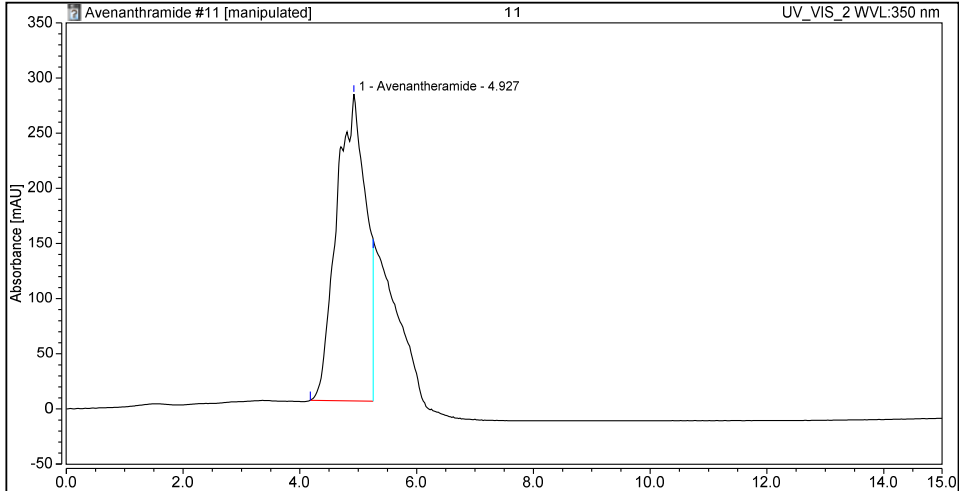 |
| HE | 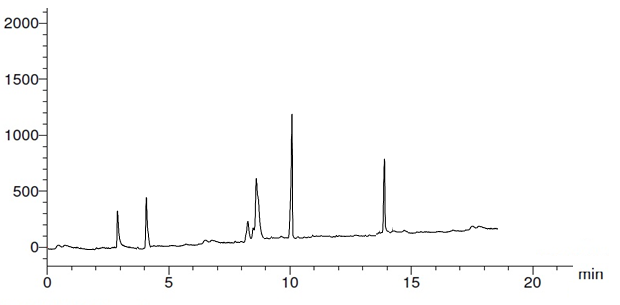 | 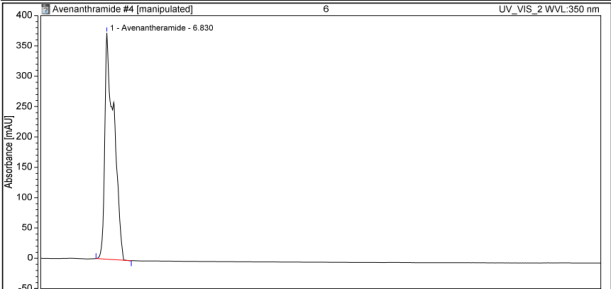 |
| ME | 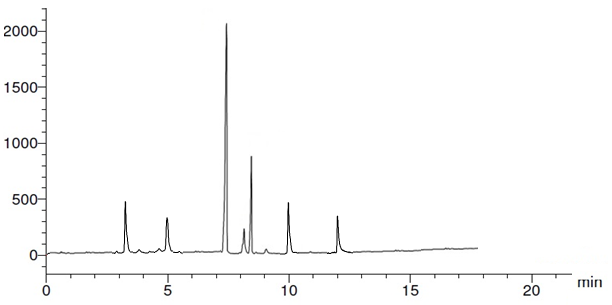 | 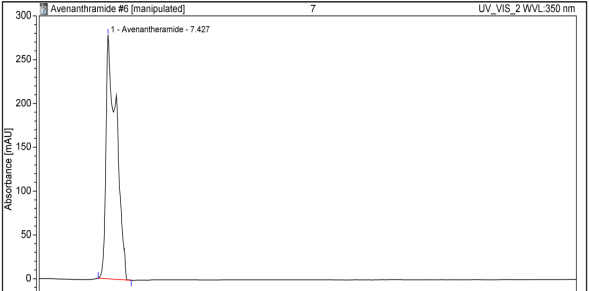 |
| AE | 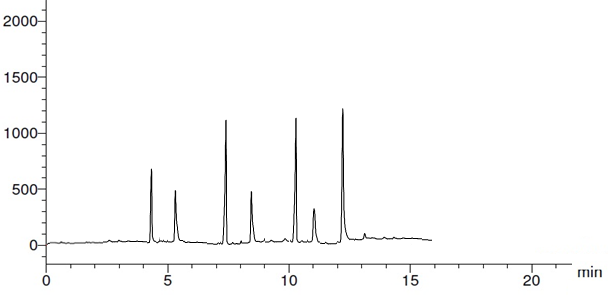 | 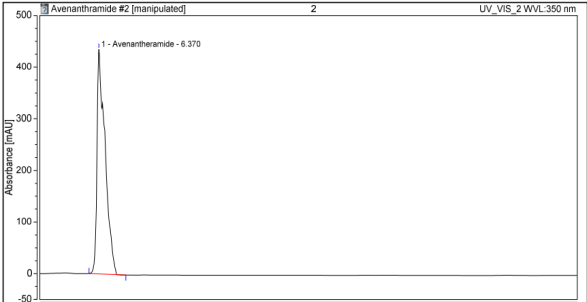 |

**Figure S8.** Effect of foliar applications (a@Cu-BTC, b@Cu-BTC, and c@Cu-BTC) on phenolic acids, and avenantheramide-C content in oat grains under calcareous soil conditions

| Treatments | Phenolic acids by HPLC | Avenantheramide-C |
| --- | --- | --- |
| **a@Cu-BTC** | 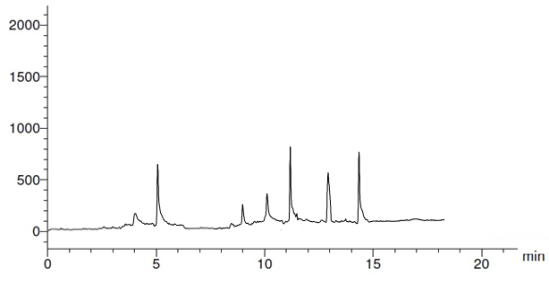 | 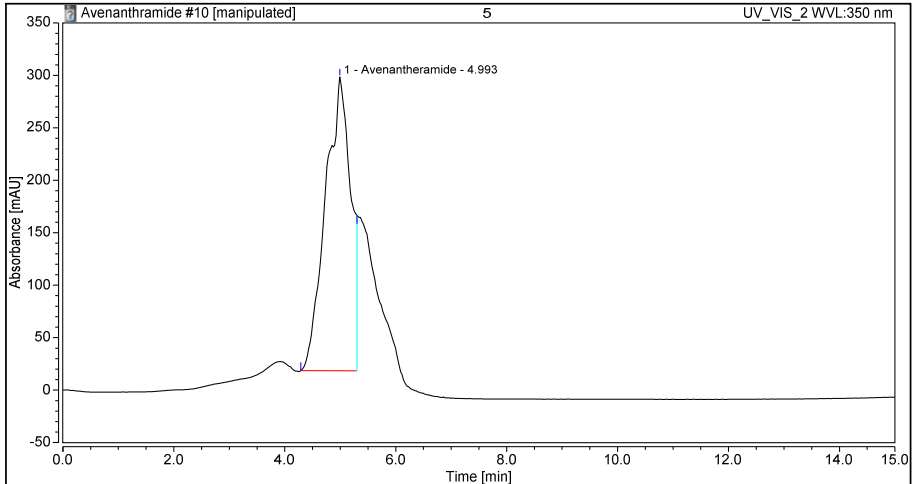 |
| **b@Cu-BTC** | 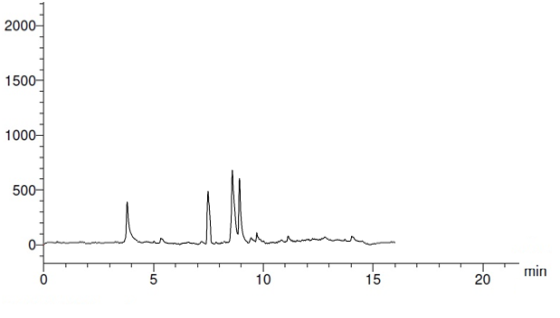 | 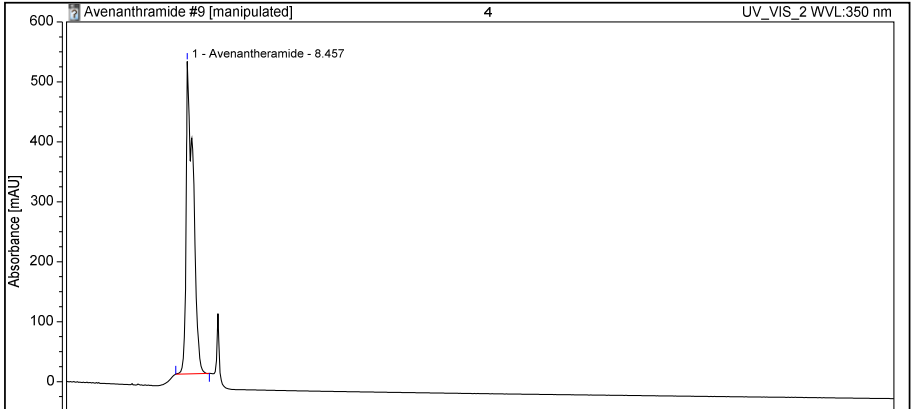 |
| **c@Cu-BTC** | 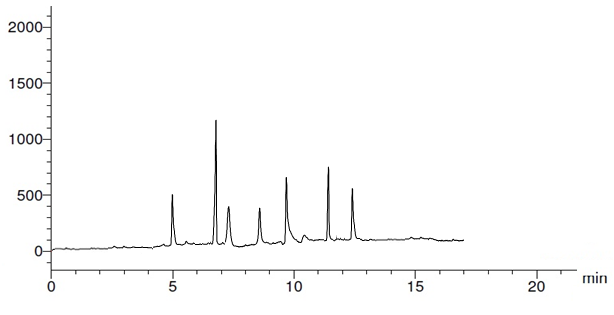 | 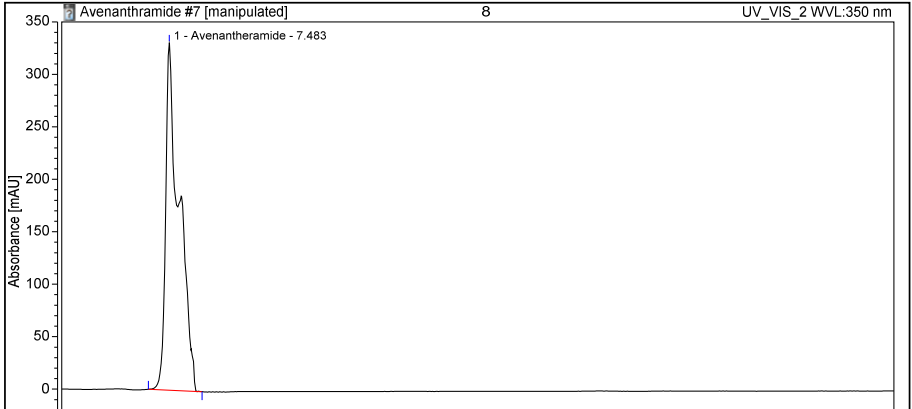 |

**Figure S9.** Effect of foliar applications (HE-a, ME-b, and AE-c) on phenolic acids, and avenantheramide-C content in oat grains under calcareous soil conditions

| Treatments | Phenolic acids by HPLC | Avenantheramide-C |
| --- | --- | --- |
| HE-a | 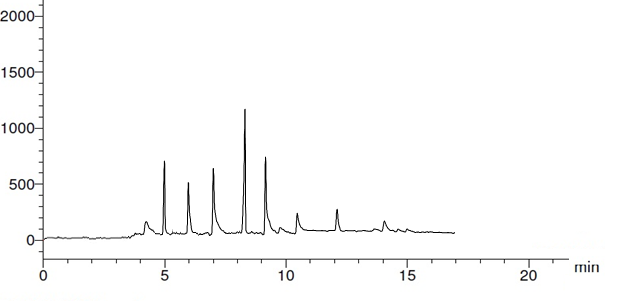 | 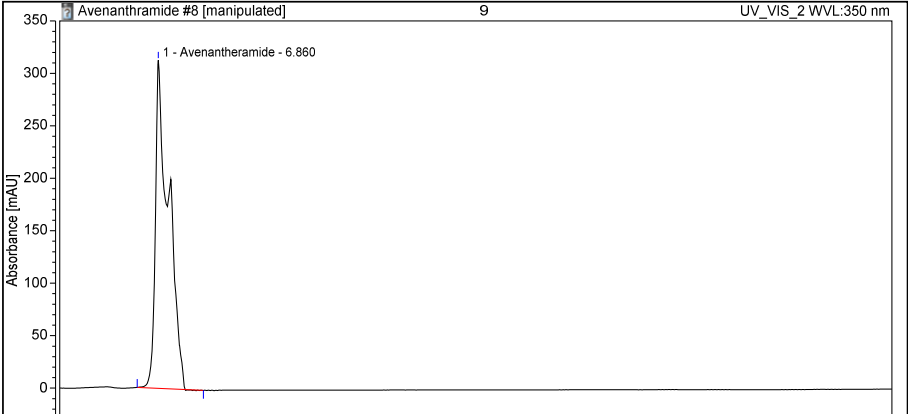 |
| ME-b | 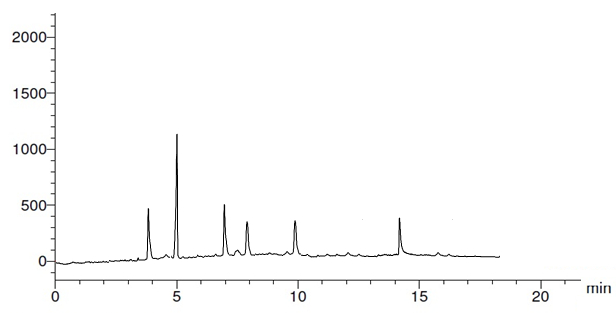 | 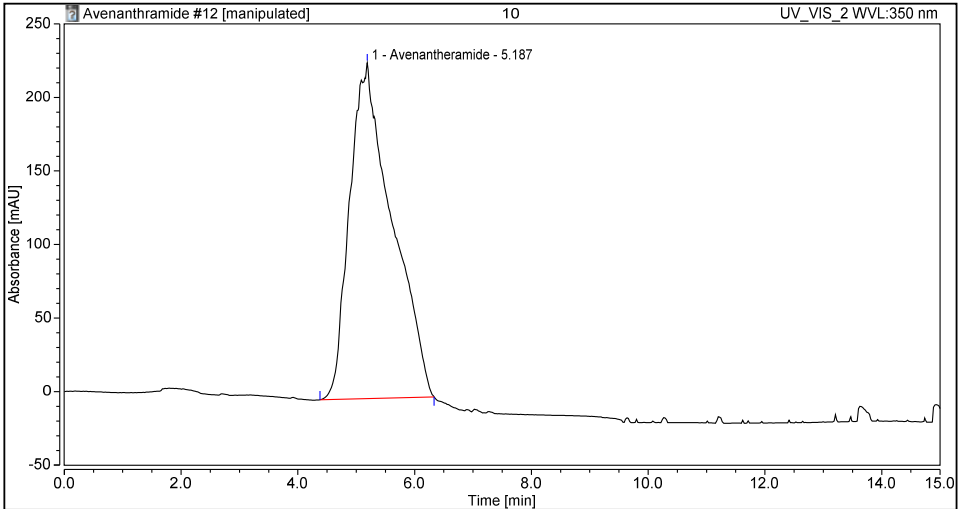 |
| AE-c | 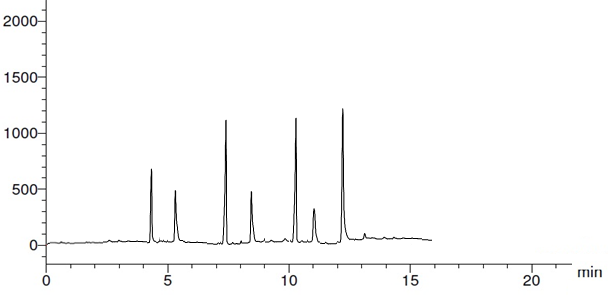 | 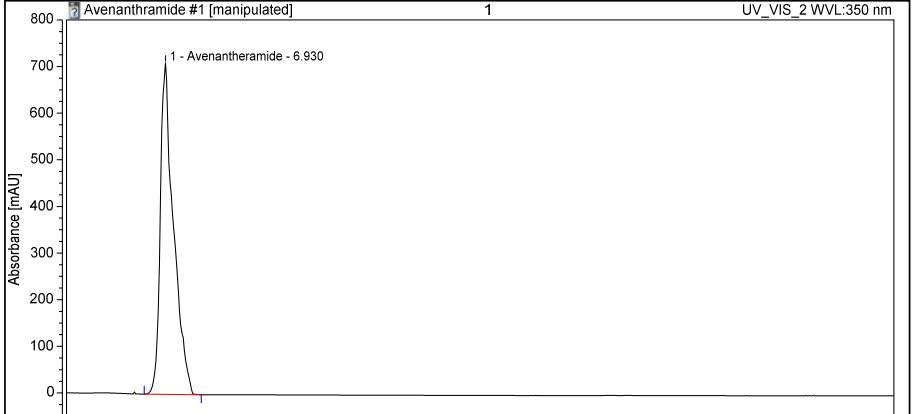 |

**Figure S10.** Effect of foliar applications (Cu-BTC, HE, ME, AE, and a@Cu-BTC) on vitamins E and K, content in oat grains under calcareous soil conditions

| Treatments | Vitamins (E & K) | Treatments | Vitamins (E & K) |
| --- | --- | --- | --- |
| Control | 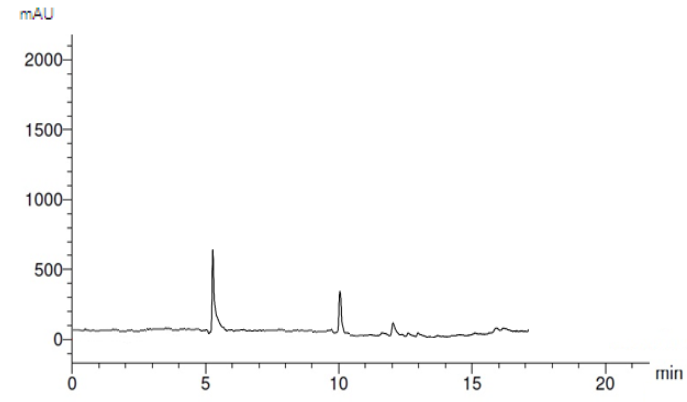 | ME | 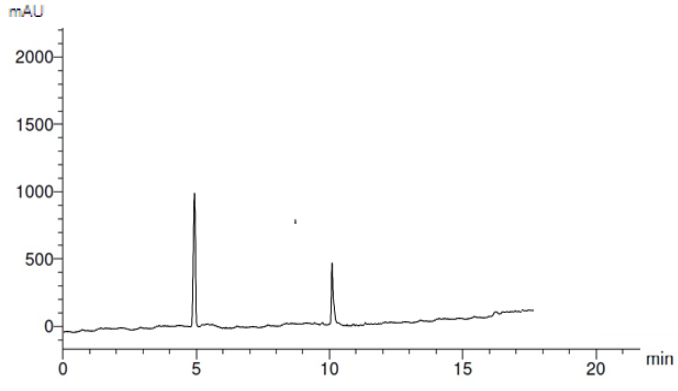 |
| Cu-BTC | 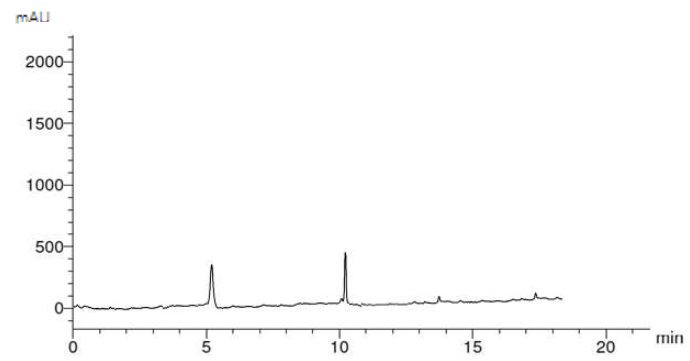 | AE | 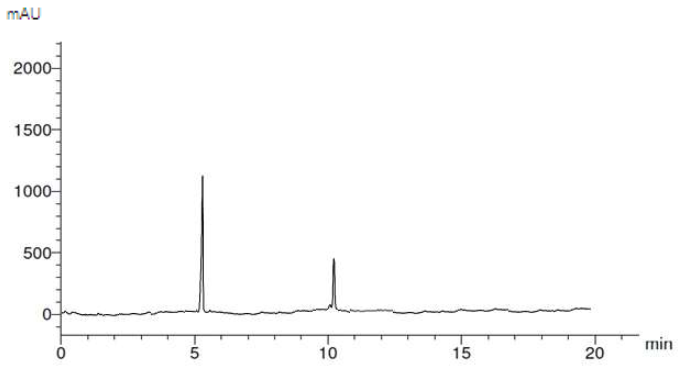 |
| HE | 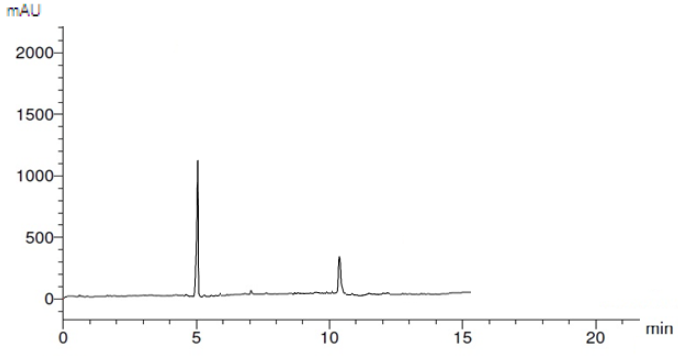 | a@Cu-BTC | 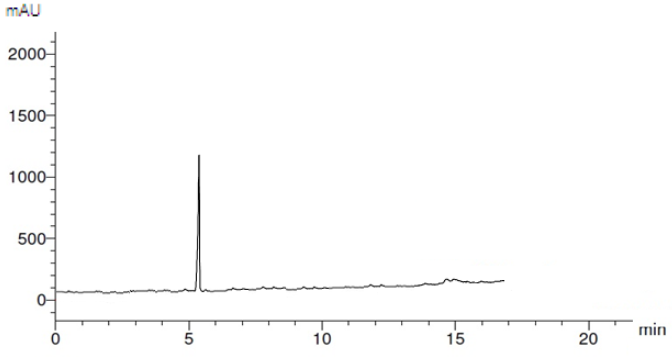 |

**Figure S11.** Effect of foliar applications (b@Cu-BTC, c@Cu-BTC, HE-a, ME-b, and AE-c) on vitamins E and K, content in oat grains under calcareous soil conditions.

| Treatments | Vitamins (E & K) | Treatments | Vitamins (E & K) |
| --- | --- | --- | --- |
| b@Cu-BTC | 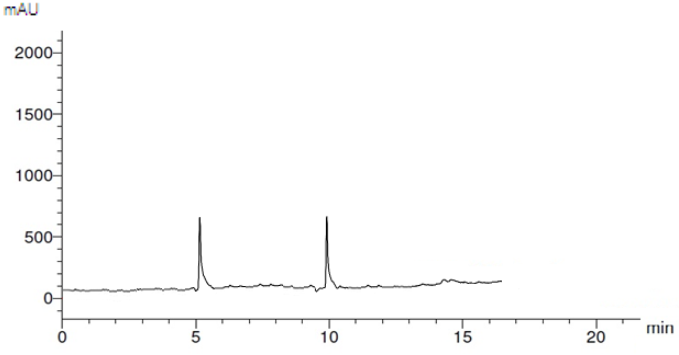 | ME-b | 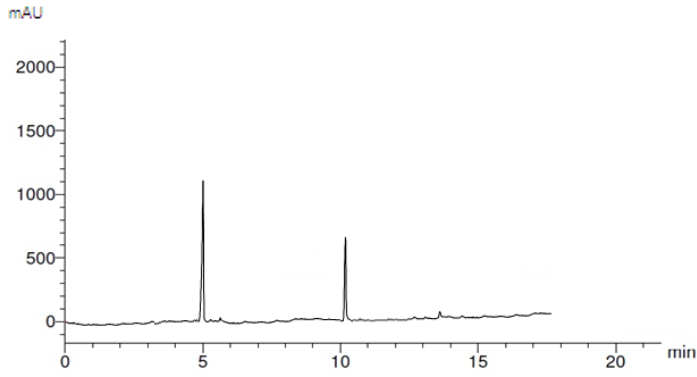 |
| c@Cu-BTC | 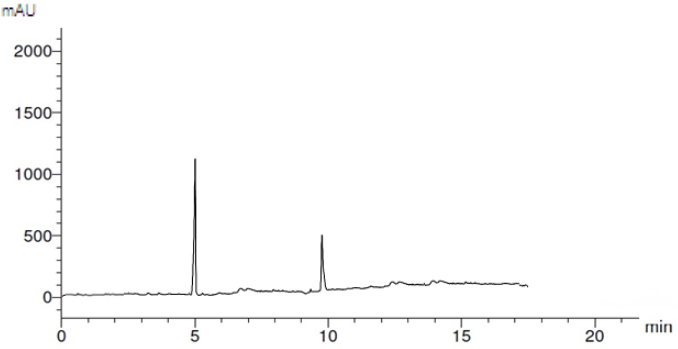 | AE-c | 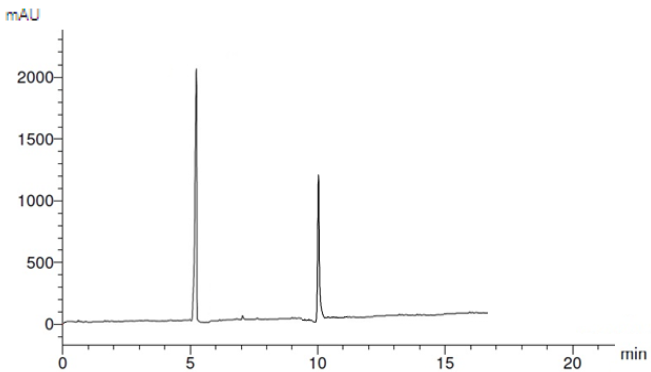 |
| HE-a | 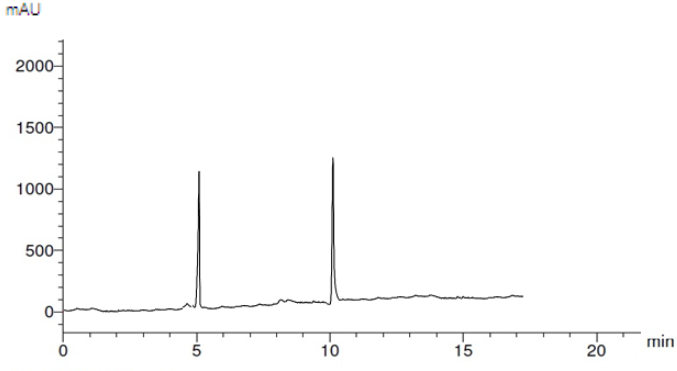 | | |

**Table S1. Chemical analysis of the experimental soil and underground irrigation water at Maryout station**

| 1. **Physical analysis of the experimental soil** | | | | | | | | | | | | | |
| --- | --- | --- | --- | --- | --- | --- | --- | --- | --- | --- | --- | --- | --- |
| **Particle size distribution %** | | | | | | | | | | | | | |
| Soil texture | | Silt and clay | | Very fine sand | | | Fine sand | | Medium sand | | Coarse sand | | Very coarse sand |
| Sandy soil | | 8.69 | | 30.22 | | | 19.06 | | 10.21 | | 12.33 | | 19.51 |
| 1. **Chemical analysis of the experimental soil** | | | | | | | | | | | | | |
| Anions (meq l^-1^) | | | | | | Cations (meq l^-1^) | | | | | pH | EC  dS m^-1^ | |
| SO_4_^--^ | Cl ^-^ | | HCO_3_ ^-^ | CO_3_^--^ | K ^+^ | | Na ^+^ | Mg^++^ | | Ca ^++^ |  |  |  |
| 4.21 | 9.31 | | 1.2 | Nil | 0.38 | | 7.83 | 3.12 | | 4.06 | 8.2 | 1.904 | |
| 1. **Chemical analysis of irrigation water** | | | | | | | | | | | | | |
| Anions (meq l^-1^) | | | | | Cations (meq l^-1^) | | | | | | pH | EC  dS m^-1^ | |
| SO_4_^--^ | Cl ^-^ | | HCO_3_ ^-^ | CO3 ^--^ | K ^+^ | | Na ^+^ | Mg ^++^ | | Ca ^++^ |  |  |  |
| 14.4 | 27.5 | | 4.1 | Nil | 0.3 | | 25.2 | 12.7 | | 9.8 | 7.0 | 4.81 | |

**Table S2. The meteorological data at Maryout site**.

| **Months** | **Parameters** | | | | | |
| --- | --- | --- | --- | --- | --- | --- |
|  | **Temp.** | **Max. Temp.** | **Min. Temp** | **RH** | **Rain** | **Wind speed** |
|  | **°C** | **°C** | **°C** | **%** | **mm** | **m/s** |
| **First season (2019/2020)** | | | | | | |
| **October** | 24.80 | 28.50 | 21.10 | 70.20 | 10.57 | 3.21 |
| **November** | 21.40 | 24.10 | 18.70 | 73.10 | 55.00 | 3.75 |
| **December** | 16.30 | 19.50 | 13.20 | 74.50 | 26.10 | 3.08 |
| **January** | 13.80 | 17.40 | 10.20 | 71.50 | 45.00 | 3.54 |
| **February** | 16.60 | 21.10 | 12.10 | 70.20 | 14.40 | 3.32 |
| **March** | 17.90 | 22.40 | 13.40 | 66.30 | 3.10 | 3.75 |
| **April** | 22.00 | 27.60 | 16.30 | 65.00 | 2.10 | 3.27 |
| **Second season (2020/2021)** | | | | | | |
| **October** | 24.50 | 27.70 | 21.10 | 70.80 | 10.00 | 3.21 |
| **November** | 20.30 | 24.90 | 16.20 | 66.00 | 24.10 | 3.11 |
| **December** | 14.80 | 18.00 | 11.40 | 70.80 | 60.00 | 3.78 |
| **January** | 12.20 | 16.40 | 8.40 | 69.10 | 49.99 | 3.30 |
| **February** | 13.40 | 18.50 | 9.30 | 72.00 | 12.38 | 3.07 |
| **March** | 15.90 | 20.90 | 11.70 | 69.00 | 2.80 | 4.04 |
| **April** | 18.40 | 23.80 | 13.10 | 64.40 | 1.60 | 4.07 |

**Abbreviations: Temp: temperature, Max: maximum, Min: minimum, RH: relative humidity**

**References**

**Aoac**. (1990). Association of official analytical chemists. Official methods of analysis. AOAC Arlington, VA.

**Booth SL, Davidson KW, Sadowski JA**. 1994. Evaluation of an HPLC method for the determination of phylloquinone (vitamin K1) in various food matrixes. *Journal of Agricultural and Food Chemistry* **42**, 295-300.

**Dubois M, Gilles KA, Hamilton JK, Rebers Pt, Smith F**. 1956. Colorimetric method for determination of sugars and related substances. *Analytical chemistry* **28**, 350-356.

**Horwitz W, Chichilo P, Reynolds H**. 1970. Official methods of analysis of the Association of Official Analytical Chemists. *Official methods of analysis of the Association of Official Analytical Chemists.*

**Kerepesi I, Toth M, Boross L**. 1996. Water-soluble carbohydrates in dried plant. *Journal of Agricultural and Food Chemistry* **44**, 3235-3239.

**Labadie MP, Boufford CE**. 1988. Gas chromatographic assay of supplemental vitamin E acetate concentrates: Collaborative study. *Journal of the Association of Official Analytical Chemists* **71**, 1168-1171.

**Magomya A, Kubmarawa D, Ndahi J, Yebpella G**. 2014. Determination of plant proteins via the kjeldahl method and amino acid analysis: A comparative study. *International journal of scientific & technology research* **3**, 68-72.

**Stratil P, Klejdus B, Kubáň V**. 2006. Determination of total content of phenolic compounds and their antioxidant activity in vegetables evaluation of spectrophotometric methods. *Journal of agricultural and food chemistry* **54**, 607-616.

**Ulusoy Hİ, Acıdereli H, Tutar U**. 2017. Optimization of extraction parameters for fat soluble vitamins and major element analysis in Polygonum cognatum Meissn plant (Madimak). *Journal of the Turkish Chemical Society Section A: Chemistry* **4**, 165-178.

**Vinson JA, Hao Y, Su X, Zubik L**. 1998. Phenol antioxidant quantity and quality in foods: vegetables. *Journal of agricultural and food chemistry* **46**, 3630-3634.

**Vinson JA, Su X, Zubik L, Bose P**. 2001. Phenol antioxidant quantity and quality in foods: fruits. *Journal of agricultural and food chemistry* **49**, 5315-5321.
